# Discovery of VU6052959/BI’7927: A Selective mGlu_3_ Positive Allosteric Modulator (PAM) from an Orthogonal Benzoxazine Chemotype

**DOI:** 10.1021/acsmedchemlett.6c00318

**Published:** 2026-07-06

**Authors:** Caleb A. Jones, Renn A. Duncan, Kristen M. Gilliland, Daniel H. Haymer, Paul K. Spearing, Rory A. Capstick, Bartholomew P. Roland, Paige Vinson, Marc Quitalig, Caroline Baggeroer, Natasha B. Billard, Jonathan W. Dickerson, Katherine J. Watson, Valerie M. Kramlinger, Olivier Boutaud, Daniel Ursu, Carsten T. Wotjak, Henning Priepke, Michel Garneau, Kristina Trenz, Colleen M. Niswender, Hyekyung P. Cho, Jerri M. Rook, Matthias Freiwald, Riccardo Giovannini, Heiko Sommer, Scott Hobson, P. Jeffrey Conn, Bruce J. Melancon, Craig W. Lindsley, Carson W. Reed

**Affiliations:** † Warren Center for Neuroscience Drug Discovery, Vanderbilt Institute for Therapeutic Advances, 5718 Vanderbilt University, Nashville, Tennessee 37232, United States; ‡ Department of Pharmacology, 12327 Vanderbilt University School of Medicine, Nashville, Tennessee 37232, United States; § Department of Chemistry, 5718 Vanderbilt University, Nashville, Tennessee 37232, United States; ∥ Vanderbilt Kennedy Center, 12328 Vanderbilt University Medical Center, Nashville, Tennessee 37232, United States; ⊥ Vanderbilt Brain Institute, 5718 Vanderbilt University, Nashville, Tennessee 37232, United States; # Vanderbilt Institute of Chemical Biology, 5718 Vanderbilt University, Nashville, Tennessee 37232, United States; ∇ Boehringer Ingelheim Pharma GmbH & Co. KG, Birkendorfer Str. 65, 88397 Biberach, Germany

**Keywords:** metabotropic glutamate receptor subtype 3, mGlu_3_, positive allosteric modulator, PAM, cognition, metabolism, novel object recognition, pharmacokinetics

## Abstract

We report the discovery of an additional selective metabotropic glutamate receptor subtype 3 (mGlu_3_) positive allosteric modulator (PAM) tool compound, VU6052959/BI’7927. A high-throughput screening (HTS) campaign identified a selective mGlu_3_ PAM VU6046180/BI’3690 (**7**) containing a benzoxazine core (an orthogonal chemotype to previously reported thiophene tool compound VU6053371/BI’8809 (**6**)). Lead optimization efforts focused on improving PK parameters led to the identification of VU6052959/BI’7927 (**8**). Orthogonal PAM **8** demonstrated efficacy in a rat novel object recognition task with conservation of our previously determined PK/PD relationship. However, an Ames positive metabolite halted further progression of **8**.

## Graphical abstract

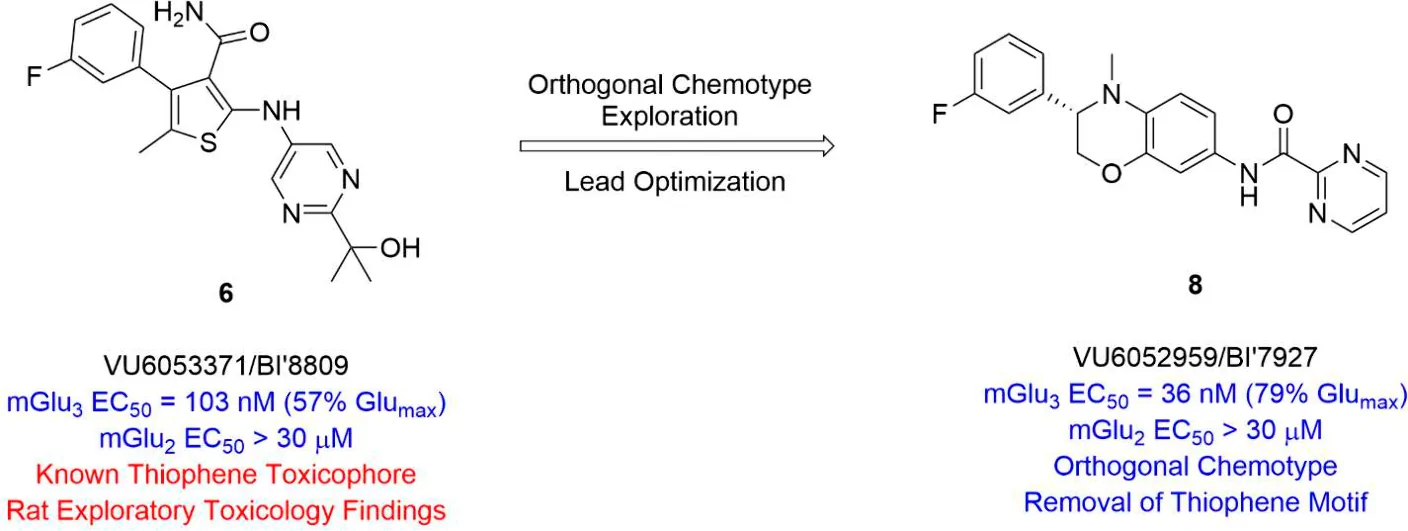

The negative symptoms and cognitive deficits observed with schizophrenia are poorly addressed by current therapeutic strategies, and these shortcomings can contribute to patient noncompliance with medication regimens.^1–3^ Current research has focused on overcoming these challenges; as a result, metabotropic glutamate receptor 3 (mGlu_3_) has generated interest as a potential therapeutic target.^4,5^ Genetic variations in the gene that encodes for mGlu_3_ (*GRM3*) are linked to schizophrenia and cognitive dysfunction.^6–10^ Activation of mGlu_3_ induces synaptic plasticity, provides neuroprotective effects, and rescues pharmacological disruptions in cognition.^10–13^ These studies utilized tool compounds **1**-**5**; however, the inclusion of a selective mGlu_3_ PAM was absent (Figure 1).^10,11^ Recently, we disclosed the discovery and optimization of the first-in-class, selective mGlu_3_ PAM tool compound **6** containing a thiophene chemotype.^14^ While PAM **6** showed efficacy in rat cognition models, the further profiling of **6** in rat exploratory toxicology studies revealed adverse neurobehavioral and clinical findings.^14^ In order to determine whether these adverse findings were chemotype-related (i.e., thiophene, a known toxicophore) or mechanism-based, reevaluation of HTS hits was prioritized in order to find an orthogonal chemical species to aid in answering this question.

**Figure 1:**
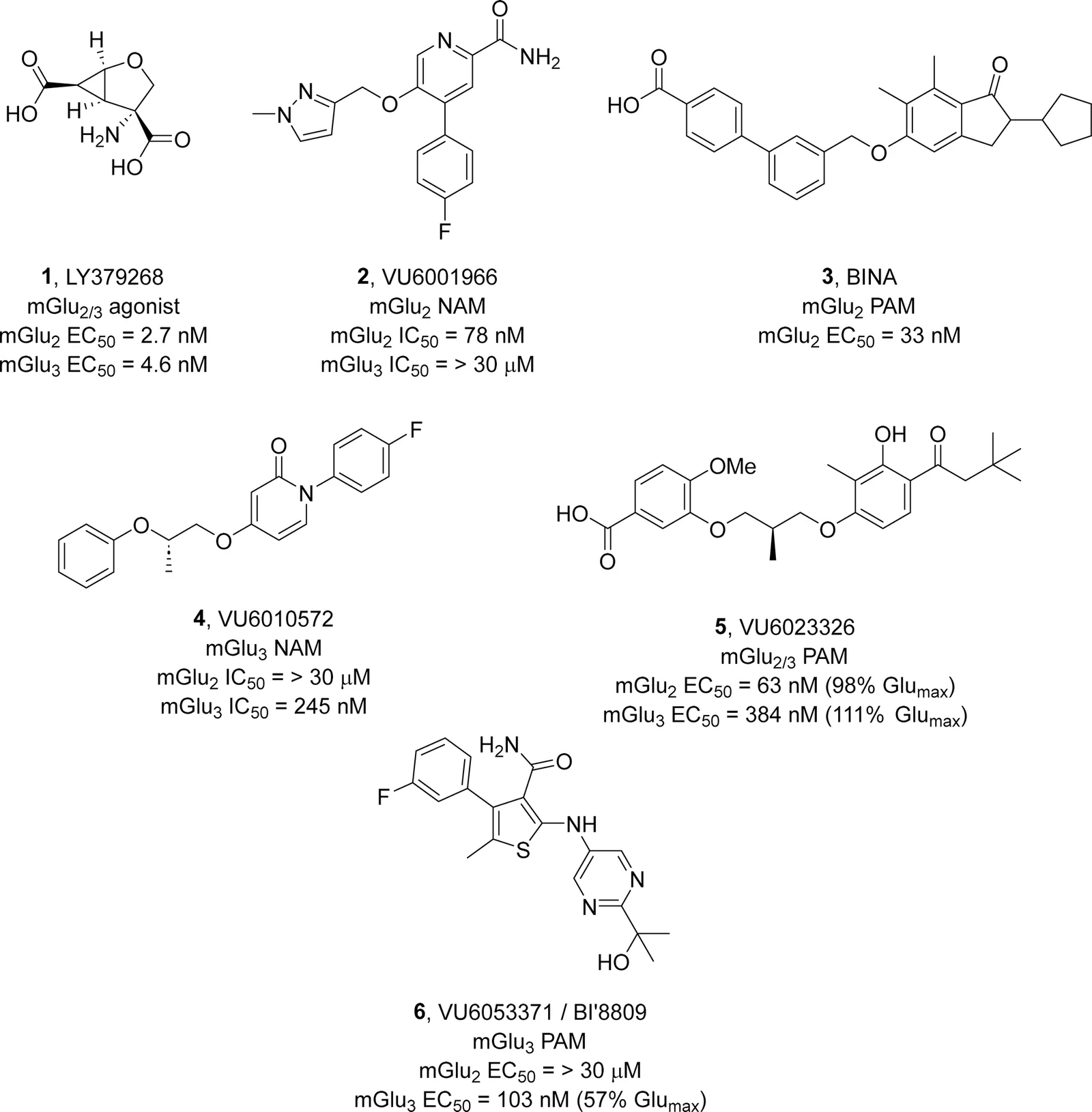
Structures of representative selective group II mGlu receptor dual orthosteric mGlu_2/3_ agonist LY379268 (**1**), selective mGlu_2_ negative allosteric modulator (NAM) VU6001966 (**2**), selective mGlu_2_ PAM BINA (**3**), selective mGlu_3_ NAM VU601576 (**4**), dual mGlu_2/3_ PAM VU6023326 (**5**), and selective mGlu_3_ PAM VU6053371/BI’8809 (**6**).

From this exercise, HTS hit VU6046180/BI’3690 (**7**) was identified (Figure 2). In a cAMP assay, **7** was a human mGlu_3_ PAM (EC_50_ = 323 nM, 35% Glu_max_) and was inactive on human mGlu_2_ (cAMP EC_50_ > 30 μM). In a receptor-regulated GIRK assay (assessing thallium flux as a surrogate for mGlu_3_ activation^15,16^ ) the selective mGlu_3_ PAM activity confirmed (GIRK human mGlu_3_ EC_50_ = 835 nM, 44% Glu_max_; GIRK human mGlu_2_ EC_50_ > 30 μM). Thus, we identified a selective mGlu_3_ PAM from an orthogonal chemical series. The structure of the benzoxazine core enabled rapid, parallel iterative synthesis of analogs en route to optimization of potency and PK properties.

**Figure 2:**
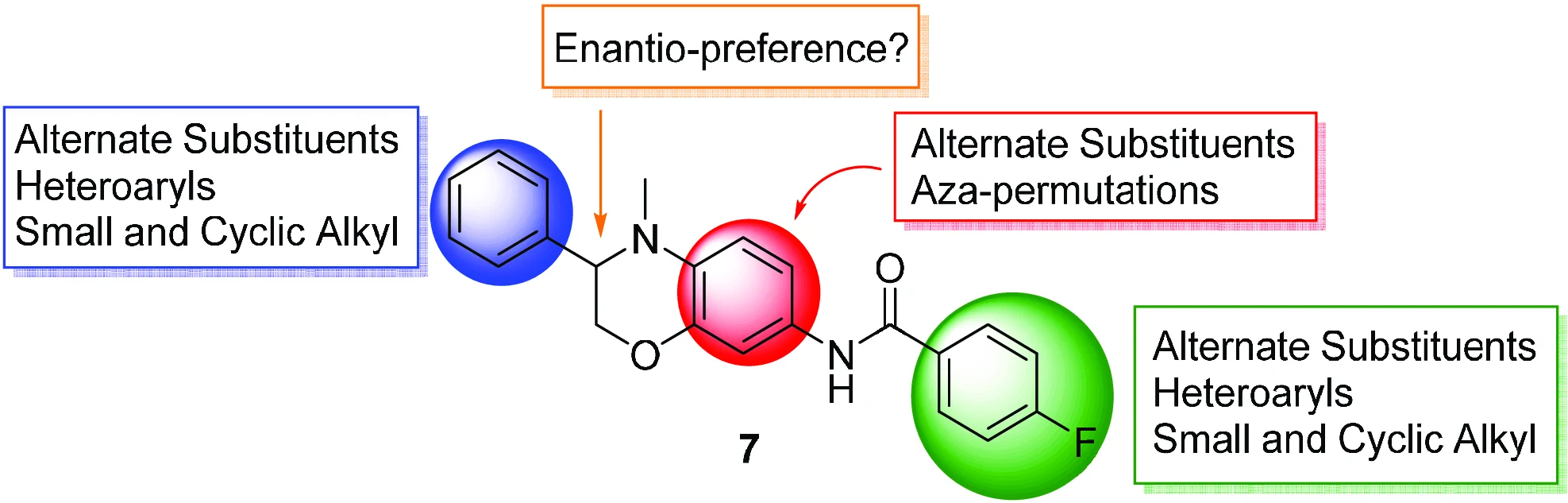
Multidimensional chemical lead optimization plan for assessing SAR of different regions of the benzoxazine scaffold.

We envisioned a multipronged approach for the chemical optimization of PAM **7** (Figure 2). Through extensive analog generation and SAR analysis (**Table S1**, see Supporting Information), it was discovered that a 3-fluorophenyl motif on the western portion of the molecule was optimal while aza-heterocycles on the eastern aromatic ring were well tolerated. Through these synthetic exercises, the two enantiomers **8** and **9** were identified (Figure 3). Gratifyingly, an enantio-preference was observed, as **8** was discovered to be a potent mGlu_3_ PAM (human mGlu_3_ GIRK EC_50_ = 36 nM, 79% Glu_max_) while **9** was less active (human mGlu_3_ GIRK EC_50_ = 2.5 μM, 52% Glu_max_). Initially prepared via a chiral supercritical fluid chromatography (SFC) separation of pure racemic material, chiral synthetic routes were developed to unambiguously assign the absolute stereochemistry of the benzoxazine ring along with optimized protocols to deliver gram-scale amounts of **8** to support additional *in vitro* and *in vivo* profiling (Scheme 1).

**Figure 3:**
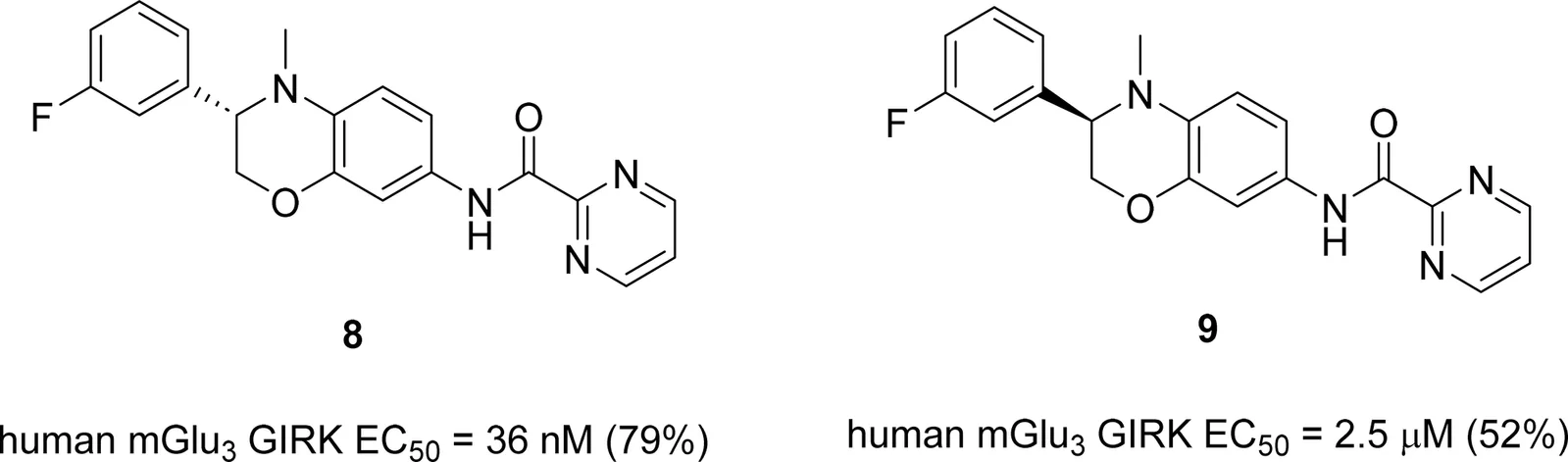
Structure and *in vitro* pharmacology data for enantiomers **8** and **9**.

**Scheme 1:**
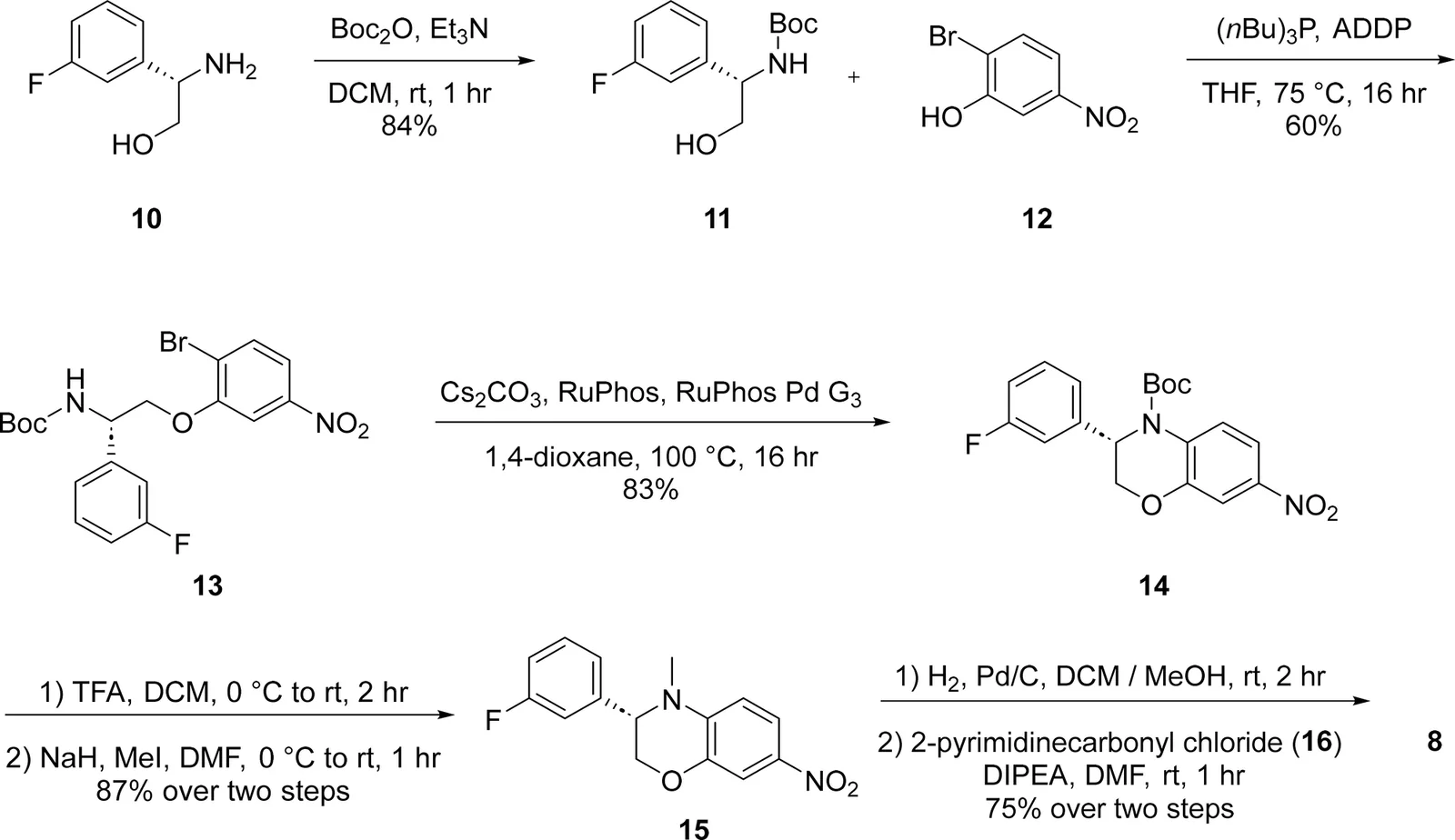
Optimized Chiral Synthetic Route for **8**

Chiral amino alcohol **10** was protected with Boc anhydride to afford alcohol **11**, which was then reacted with phenol **12** in a Mitsunobu reaction employing ADDP and tributylphosphine ((*n*Bu)_3_P) to produce intermediate **13**.^17^ An intramolecular Buchwald-Hartwig coupling furnished Boc-protected benzoxazine **14**.^18^ Subsequent Boc-deprotection and *N*-methylation yielded nitro-benzoxazine **15** in excellent yield over two steps. Lastly, nitro reduction followed by immediate acylation of the resulting aniline intermediate with 2-pyrimidinecarbonyl chloride **16** afforded **8** in quantities sufficient for additional *in vitro* and *in vivo* profiling.

**8** was a mGlu_3_ PAM for both human (EC_50_= 36 nM, 79% Glu_max_) and rat mGlu_3_ (EC_50_= 16 nM, 65% Glu_max_). Importantly, **8** was inactive (EC_50_ > 30 μM) at both human and rat mGlu_2_, mGlu_1_, and mGlu_5_. PAM **8** displayed good free fraction in both plasma (human and rat *f*_u_ = 0.04 and 0.04, respectively) and rat brain (BHB *f*_u_ = 0.01). The predicted hepatic clearance of **8** was also found to be favorable (CL_hep_ (h,r) = 5.7 and 24.9 mL/min/kg, respectively). Additionally, **8** displayed a good CYP_450_ profile (IC_50_s: 3A4 (>30 μM), 2D6 (>30 μM), 2C9 (16 μM), 1A2 (>30 μM)). **8** possessed good kinetic aqueous solubility (65 μM at pH = 6.8; 70 μM at pH = 2.2), and high solubility in both FaSSIF (84 μg/mL) and FaSSGF (30 μg/mL). Predicted human CNS penetration in an MDCK-MDR1 assay for **8** was high (P-gp ER = 0.4; P_app_ = 62 × 10^–6^ cm/s). In an ancillary pharmacology panel (Eurofins Lead Profiling Screen), displacement of radioligands were observed at human dopamine transporter (DAT) (63% at 10 μM) and human norepinephrine transporter (NET) (66% at 10 μM).^15^ Subsequent radioligand binding assays for DAT produced a binding IC_50_ of 3.9 μM and a K_i_ of 3.1 μM (affording at least an ∼ 100-fold selectivity window).^15^ Additionally, PAM **8** displayed an acceptable *in vivo* pharmacokinetic profile. In a rat IV/PO PK crossover study, **8** exhibited low clearance (CL_p_ = 19.8 mL/min/kg), as well as moderate half-life (*t*_1/2_ = 0.8 h), moderate mean residence time (MRT = 0.9 h), good volume of distribution (Vss = 1.1 L/kg), and good oral bioavailability (%F = 60%). In a rat IV plasma:brain level (PBL) cassette (0.2 mg/kg, 15 min time point), **8** displayed robust CNS exposure (K_p_ = 2.4 and K_p,uu_ = 0.6) (Table 1).

**Table 1:**
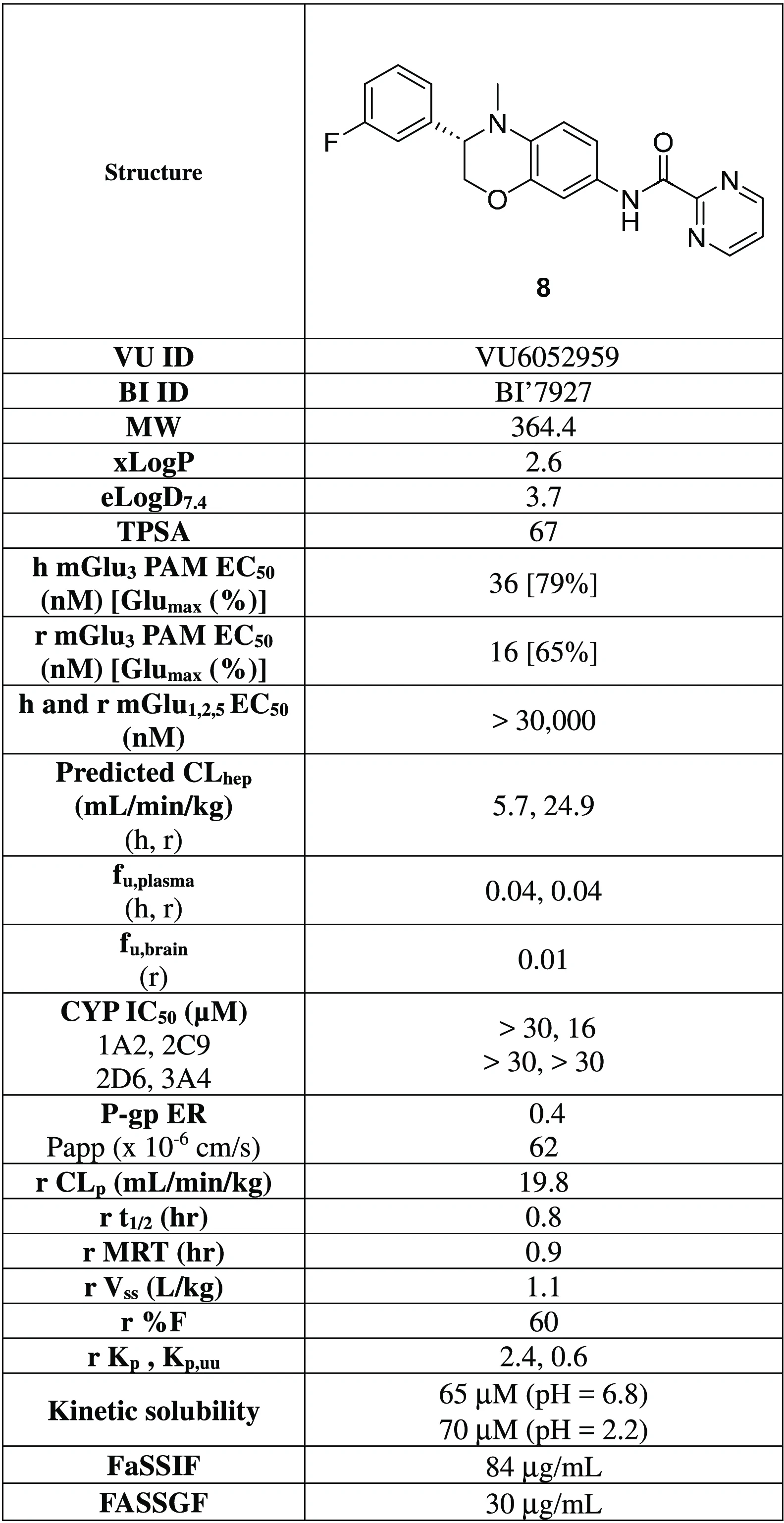
Pharmacology, *In Vitro*, and *In Vivo* DMPK Profile of **8** ^a^

a h = human and r = rat.

Similar to the previous thiophene PAM tool compound **6**, we assessed orthogonal PAM **8** in a rat novel object recognition (NOR) assay (Figure 4).^14,19^ At 10 mg/kg PO, robust enhancement in rat recognition memory was observed. At this dose, unbound brain levels of **8** were 50.1 nM (∼3.1-fold the rat mGlu_3_ PAM EC_50_). Thus, unbound brain exposures at, or above, the rat mGlu_3_ PAM EC_50_ affords a robust PK/PD relationship for efficacy in the NOR paradigm. This is in alignment with what was observed with previous thiophene tool compound **6**.^14^

**Figure 4:**
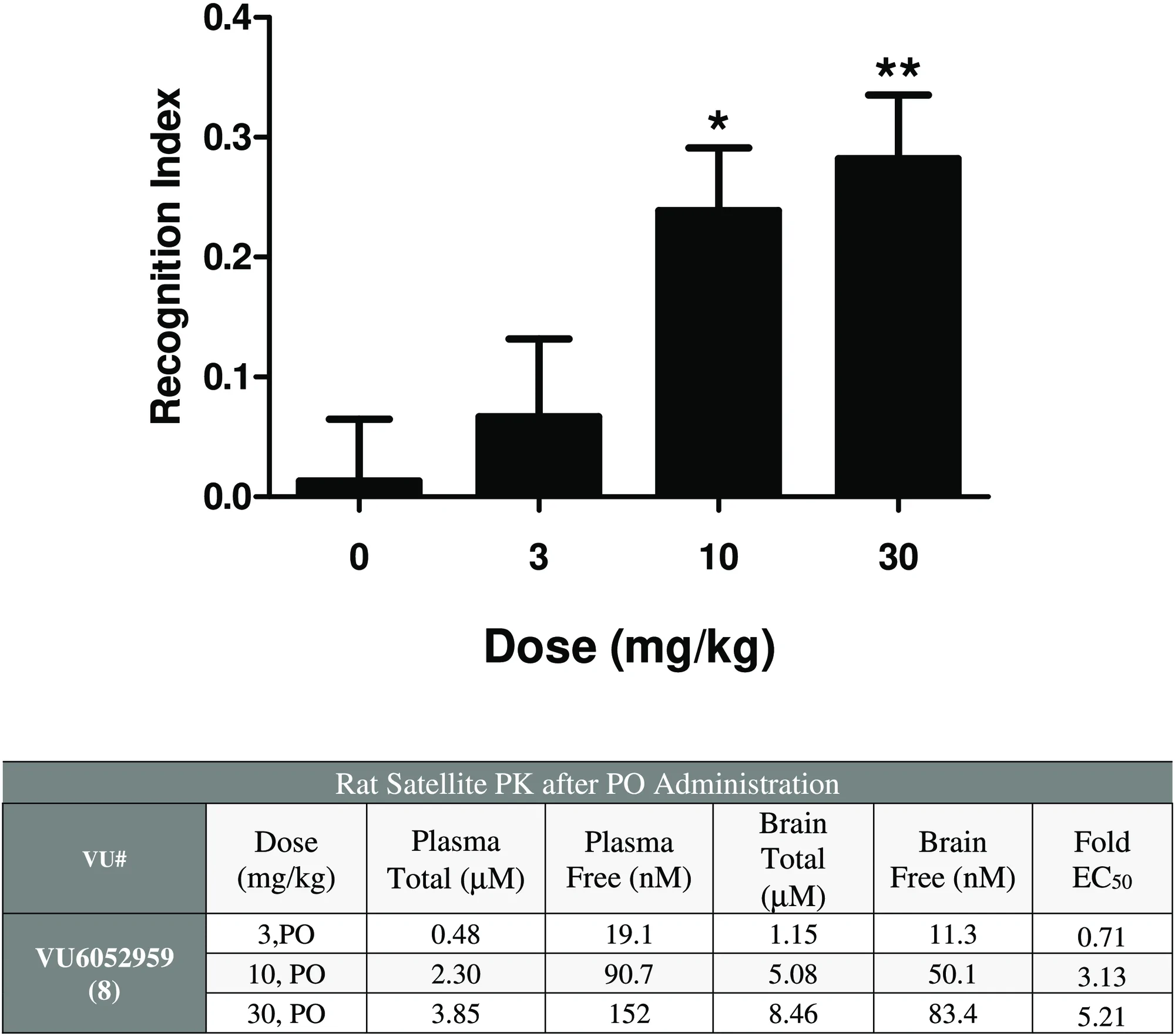
Novel Object Recognition (NOR) test in male Sprague–Dawley rats with VU6052959/BI’7927 (**8**). PAM **8** dose-dependently enhanced recognition memory in rats. Pretreatment with 3 (*N* = 20), 10 (*N* = 19) and 30 (*N* = 19) mg/kg **8** (PO, 0.5% natrosol/0.015% Tween 80 in water, 30 min) prior to exposure to identical objects significantly enhanced recognition memory assessed 24 h later. *p < 0.05; **p < 0.01 One-way ANOVA, Dunnett post hoc test.

Next, PAM **8** was assessed for its ability to reverse various pharmacological-induced disruptions in NOR. For these studies, both the NMDA receptor antagonist MK-801 (Figure 5A) and the muscarinic receptor antagonist scopolamine (Figure 5B) were chosen as the pharmacological challenges. In both cases, PAM **8** dose-dependently reversed these deficits in rat NOR. Exposure data from rat satellite PK animals correlate well with the aforementioned PK/PD relationship.

**Figure 5:**
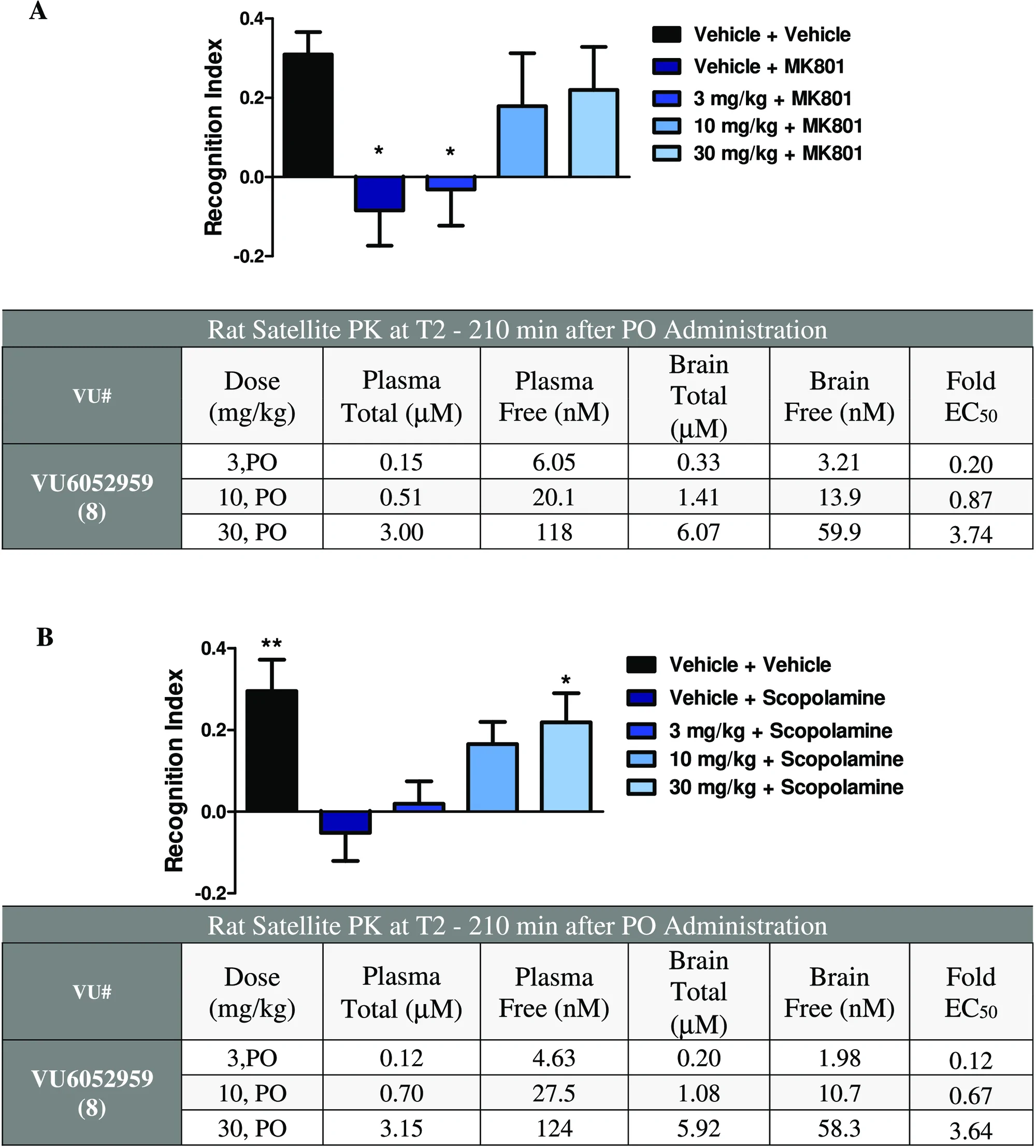
(A) MK-801-induced (0.075 mg/kg, s.c., saline) disruptions in NOR were dose-dependently reversed by PAM **8** (3, 10, and 30 mg/kg **8** PO, 0.5% natrosol/0.015% Tween 80 in water). *N* = 7–12/group of male Sprague–Dawley rats. ANOVA *p < 0.05; Dunnett post hoc test as compared to vehicle + MK-801 control. (B) Scopolamine-induced (1 mg/kg, i.p., 10% Tween 80) disruptions in NOR were dose-dependently reversed by PAM **8** (3, 10, and 30 mg/kg **8** PO, 0.5% natrosol/0.015% Tween 80 in water). *N* = 10–12/group of male Sprague–Dawley rats. ANOVA *p < 0.05; Dunnett post hoc test as compared to vehicle + scopolamine control.

To determine if mGlu_3_ engagement during the familiar object presentation (T1) is necessary to enhance NOR in rat, mGlu_3_ PAM **8** was administered at 14 or 1 h prior to T1 (30 mg/kg PO). Similar to the data in Figure 4, animals pretreated with 30 mg/kg of **8** 1 h prior to T1 exhibited significantly increased recognition memory compared to vehicle-treated control animals (Figure 6). Increasing the pretreatment time for **8** to 14 h prior to T1 significantly attenuated the enhancement of recognition memory when compared to vehicle-treated control animals (Figure 6). This indicates that target engagement during familiar object presentation (T1) is required to enhance recognition memory in these animals.

**Figure 6:**
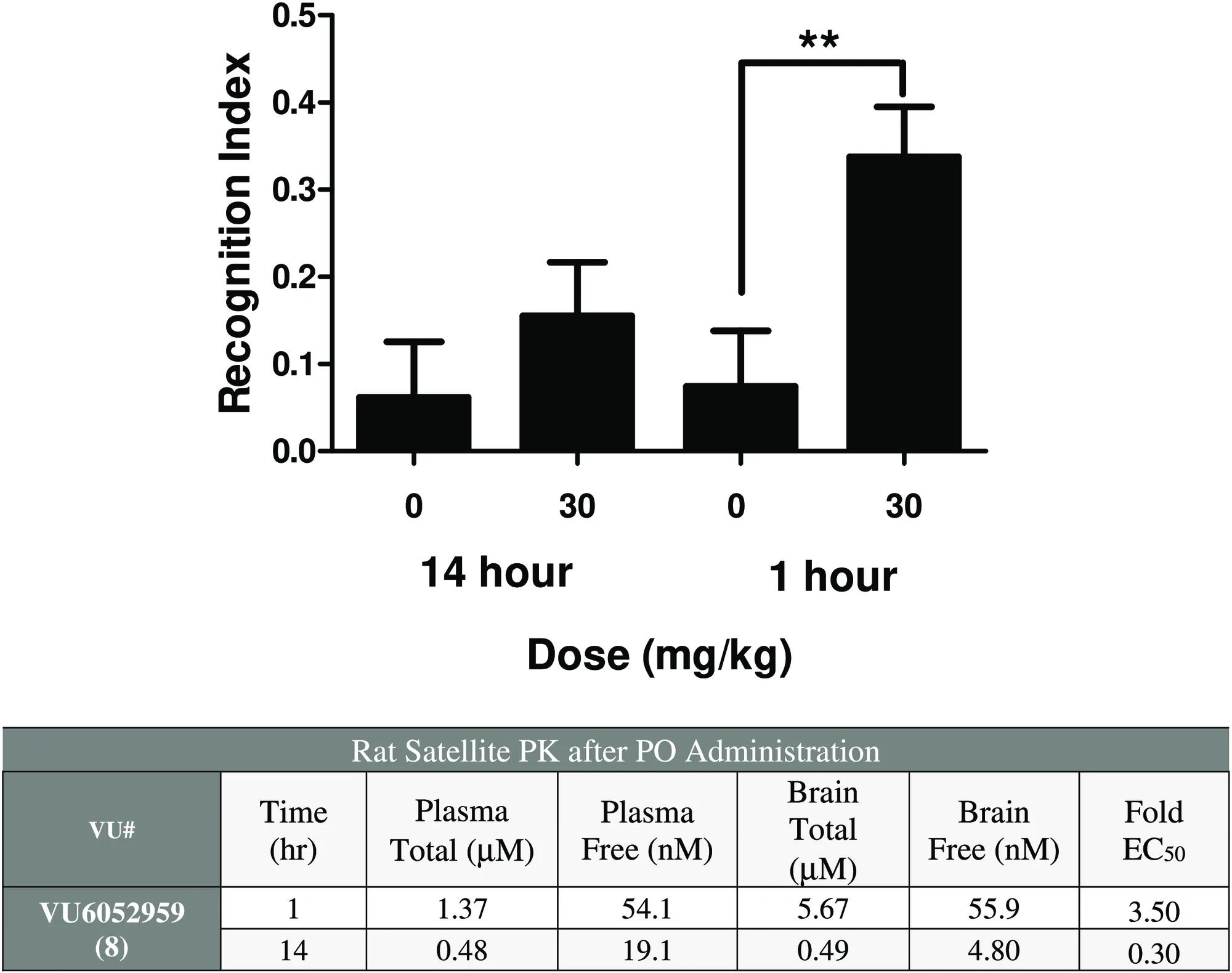
NOR test in male Sprague–Dawley rats after 14 or 1 h pretreatment with **8**. Pretreatment of 14 h with 30 mg/kg **8** did not enhance recognition index (*p* = 0.30; *N* = 15). Animals pretreated with 30 mg/kg of **8** 1 h before T1 demonstrated enhanced recognition memory when compared to vehicle control animals (**p = 0.005; unpaired *t* test; *N* = 14–15). Satellite PK data shows free brain exposure of **8** for this study.

Next, subchronic dosing of **8** was utilized to determine if repeated dosing over 5 days would result in tachyphylaxis or a priming effect of **8** on NOR. The dosing paradigm was performed as shown in Figure 7 and was divided into 5 groups (Group I received vehicle only, Group II received **8** 1 h prior to T1, Group III received **8** 24 h prior to T1, Group IV received **8** daily for 5 days prior to T1 and 1 h prior to T1, and Group V received **8** daily for 5 days prior to T1 with the last dose 24 h prior to T1). The animals in Group III and V, where the last dose of **8** was 24 h prior to T1, did not demonstrate a significant enhancement of recognition index when compared to Group I (vehicle control animals; Figure 7). The animals in Groups II and IV, in which the final or only dose of **8** was administered 1 h prior to T1, demonstrated enhanced recognition index when compared to the Group I control animals (Figure 7). There was no significant difference in the recognition index for animals in Group II when compared to Group IV. This indicates that **8** does not exhibit priming or tachyphylaxis in this assay and further demonstrates that sufficient extent of target engagement by **8** at T1 is required to enhance recognition memory in these animals.

**Figure 7:**
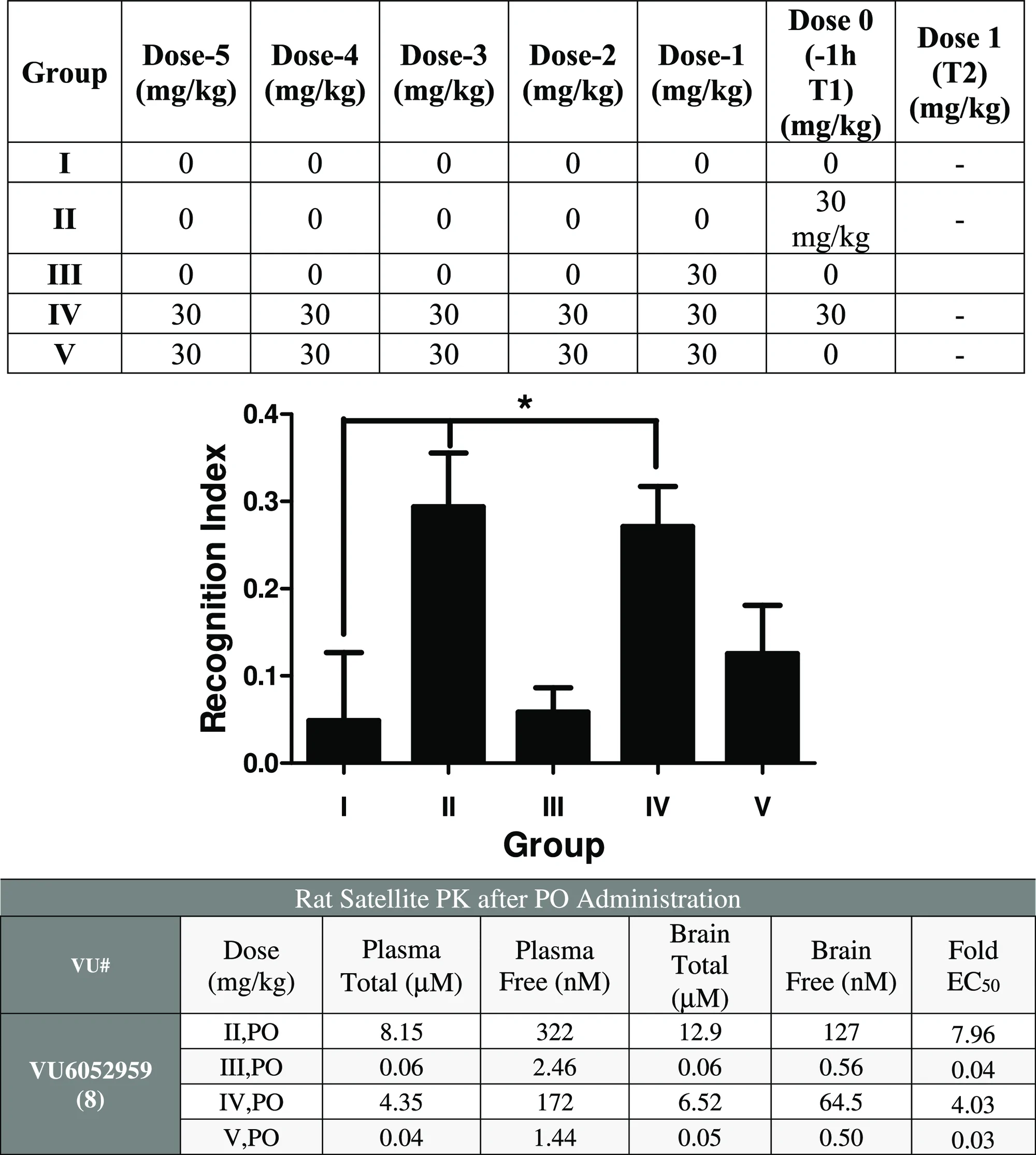
NOR test in male Sprague–Dawley rats after subchronic pretreatment with **8**. Animals pretreated 1 h before T1 with 30 mg/kg of **8** demonstrated enhanced recognition memory when compared to vehicle control animals (Group II compared to Group I; *p < 0.05; One-way ANOVA with Dunnett’s Multiple Comparison test; *N* = 12–14). Similarly, animals pretreated daily for 5 days with 30 mg/kg of **8** and then 1 h prior to T1 exhibited an enhanced recognition index compared to vehicle treated control animals (Group IV vs Group 1; *p < 0.05; *N* = 13–14). Animals pretreated with 30 mg/kg of **8** 24 h prior to T1 did not show enhanced recognition memory compared to vehicle-treated animals (Group III vs Group I; *N* = 13–14). Similarly, daily dosing of 30 mg/kg of **8** for 5 days with the last dose 24 h prior to T1 did not enhance recognition index compared to vehicle-treated controls (Group V vs Group I; *N* = 13–14).

We next assessed the well-established metabolic liability of the amide linker within PAM **8**, as upon potential amide hydrolysis, electron-rich benzoxazine aniline **17** would be released (Scheme 2). Since electron-rich anilines are known structural alerts/toxicophores, it was paramount that this potential metabolite **17** be tested in an Ames assay (both with and without metabolic activation) to assess its mutagenic potential. Unfortunately, electron-rich aniline **17** produced an Ames positive result, precluding further development of **8** as a candidate.

**Scheme 2:**
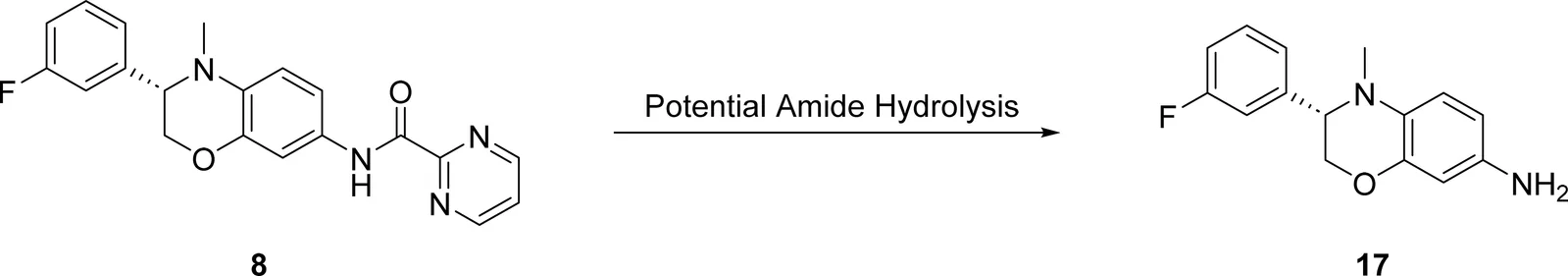
Potential Amide Hydrolysis of **8** Would Liberate Electron-Rich Aniline **17**

In summary, the discovery of VU6052959/BI’7927 (**8**), a selective, CNS-penetrant mGlu_3_ PAM from an orthogonal chemical class is disclosed. PAM **8** was found to be a robust *in vivo* tool compound with efficacy in rat NOR assays. However, electron-rich aniline **17** (a potential amide hydrolysis metabolite) produced an Ames positive result which led to discontinuation of **8**. Progress in rectifying these issues is ongoing and will be reported in due course.

## Supplementary Material



## Notes

Additional experimental details, methods for the synthesis and characterization of all compounds, *in vitro* and *in vivo* DMPK protocols, and supplemental tables. All final compounds are >95% pure by HPLC and ^1^H NMR analysis. (PDF)
