# Supplementary material for: Discovery of VU6052959/BI’7927: A Selective mGlu3 Positive Allosteric Modulator (PAM) from an Orthogonal Benzoxazine Chemotype
Source: ACS Med Chem Lett. 2026 Jul 6;17(8):1901–8. doi: 10.1021/acsmedchemlett.6c00318 (PMC13488376; doi:10.1021/acsmedchemlett.6c00318)
Supplement: Supplementary file 1 [file ml6c00318_si_001.pdf]

Discovery of VU6052959/BI’7927: a Selective mGlu$_3$ Positive Allosteric Modulator (PAM) From an Orthogonal Benzoxazine Chemotype

Caleb A. Jones,[1,2] Renn A. Duncan,[1,2] Kristen M. Gilliland,[1,2] Daniel H. Haymer,[1,2] Paul K. Spearing,[1,2] Rory A. Capstick,[1,2] Bartholomew P. Roland,[1,2] Paige Vinson,[1,2] Marc Quitalig,[1,2] Caroline Baggeroer,[1,2]Natasha B. Billard,[1,2] Jonathan W. Dickerson,[1,2] Katherine J. Watson,[1,2] Valerie M. Kramlinger,[1,2] Olivier Boutaud,[1,2] Daniel Ursu,[7] Carsten T. Wotjak,[7] Henning Priepke,[7] Michel Garneau,[7] Kristina Trenz,[7], Colleen M. Niswender,[1,2,4,5,6] Hyekyung P. Cho,[1,2] Jerri M. Rook,[1,2,5] Matthias Freiwald,[7] Riccardo Giovannini,[7] Heiko Sommer,[7] Scott Hobson,[7] P. Jeffrey Conn,[1,2] Bruce J. Melancon[1,2] Craig W. Lindsley[1,2,3,6], and Carson W. Reed*[1,2]

Affiliation:
[1]Warren Center for Neuroscience Drug Discovery, Vanderbilt Institute for Therapeutic Advances, Vanderbilt University, Nashville, TN 37232, USA
[2]Department of Pharmacology, Vanderbilt University School of Medicine, Nashville, TN 37232, USA
[3]Department of Chemistry, Vanderbilt University, Nashville TN 37232, USA
[4]Vanderbilt Kennedy Center, Vanderbilt University Medical Center, Nashville, TN 37232, USA
[5]Vanderbilt Brain Institute, Vanderbilt University, Nashville, TN 37232, USA
[6]Vanderbilt Institute of Chemical Biology, Vanderbilt University, Nashville TN 37232, USA
[7]Boehringer Ingelheim Pharma GmbH & Co. KG, Birkendorfer Str. 65, 88397 Biberach, Germany

*To whom correspondence should be addressed at carson.w.reed@vanderbilt.edu

Table of Contents

General synthetic methods and instrumentation……………………………………………………S2

Synthesis of HTS Hit **7**…………………………………………………………………………..S3

Table S1. SAR table of select examples………………………………………………………S6

Chiral SFC separation conditions for **8** and **9**……………………………………………………S9

Chiral synthesis of **8**………………………………………………………………………………S13

Table S2. Eurofins lead profiling screen data for **8**…………………………………...……......S17

In vitro molecular pharmacology methods (cAMP)…..………………………………………..S19

In vitro molecular pharmacology methods (GIRK)…..………………………………..……..S23

In vitro and *in vivo* DMPK methods……………………………………………………………S25

Behavioral pharmacology methods……………………………………………………………S29

General Synthetic Methods and Instrumentation

All NMR spectra were recorded on a 400 MHz AMX Bruker NMR spectrometer. ^{1}H and ^{13}C chemical shifts are reported in δ values in ppm downfield with the deuterated solvent as the internal standard. Data are reported as follows: chemical shift, multiplicity (s = singlet, d = doublet, t = triplet, q = quartet, b = broad, m = multiplet), integration, coupling constant (Hz). Low resolution mass spectra were obtained on an Agilent 6120 or 6150 with ESI source. MS parameters were as follows: fragmentor: 70, capillary voltage: 3000 V, nebulizer pressure: 30 psig, drying gas flow: 13 L/min, drying gas temperature: 350 ºC. Samples were introduced via an Agilent 1290 UHPLC comprised of a G4220A binary pump, G4226A ALS, G1316C TCC, and G4212A DAD with ULD flow cell. UV absorption was generally observed at 215 nm and 254 nm with a 4 nm bandwidth. Column: Waters Acquity BEH C18, 1.0 x 50 mm, 1.7 um. Gradient conditions: 5% to 95% CH_3CN in H_2O (0.1% TFA) over 1.4 min, hold at 95% CH_3CN for 0.1 min, 0.5 mL/min, 55 ºC. High resolution mass spectra were obtained on an Agilent 6540 UHD Q-TOF with ESI source. MS parameters were as follows: fragmentor: 150, capillary voltage: 3500 V, nebulizer pressure: 60 psig, drying gas flow: 13 L/min, drying gas temperature: 275 ºC. Samples were introduced via an Agilent 1200 UHPLC comprised of a G4220A binary pump, G4226A ALS, G1316C TCC, and G4212A DAD with ULD flow cell. UV absorption was observed at 215 nm and 254 nm with a 4 nm bandwidth. Column: Agilent Zorbax Extend C18, 1.8 µm, 2.1 x 50 mm. Gradient conditions: 5% to 95% CH_3CN in H_2O (0.1% formic acid) over 1 min, hold at 95% CH_3CN for 0.1 min, 0.5 mL/min, 40 ºC. For compounds that were purified on a Gilson preparative reversed-phase HPLC, the system comprised of a 333 aqueous pump with solvent-selection valve, 334 organic pump, GX-271 or GX-281 liquid hander, two column switching valves, and a 155 UV detector. UV wavelength for fraction collection was user-defined, with absorbance at 254 nm always monitored. Method 1: Phenomenex Axia-packed Luna C18, 30 x 50 mm, 5 µm column. Mobile phase: CH_3CN in H_2O (0.1% TFA). Gradient conditions: 0.75 min equilibration, followed by user defined gradient (starting organic percentage, ending organic percentage, duration), hold at 95% CH_3CN in H_2O (0.1% TFA) for 1 min, 50 mL/min, 23 ºC. Method 2: Phenomenex Axia-packed Gemini C18, 50 x 250 mm, 10 um column. Mobile phase: CH_3CN in H_2O (0.1% TFA). Gradient conditions: 7 min equilibration, followed by user defined gradient (starting organic percentage, ending organic percentage, duration), hold at 95% CH_3CN in H_2O (0.1% TFA) for 7 min, 120 mL/min, 23 ºC.

Note: All final compounds are >95% pure by HPLC and ^{1}H NMR.

Safety statement: No unexpected or unusually high safety hazards were encountered.

Synthesis for HTS Hit 7

Step 1: Synthesis of 7-nitro-3-phenyl-3,4-dihydro-*2H*-benzo[*b*][1,4]oxazine

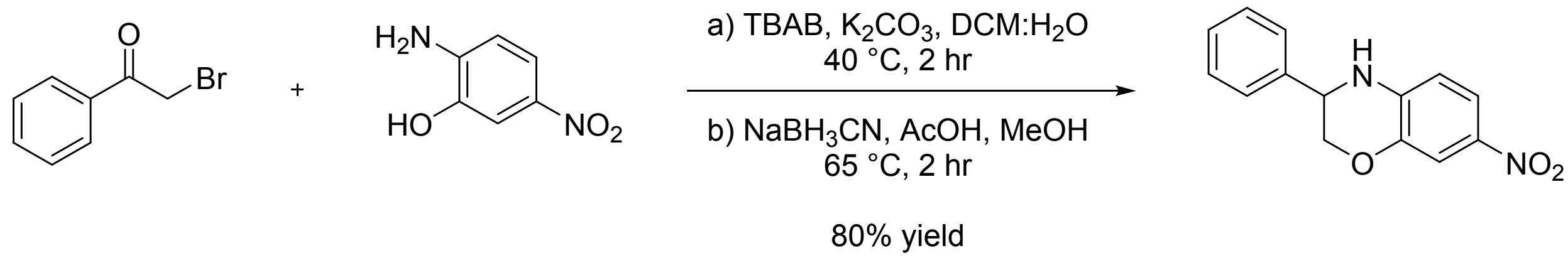

To a suspension of tetrabutylammonium bromide (TBAB) (210 mg, 0.65 mmol), 2-bromoacetophenone (646 mg, 3.25 mmol), and 2-amino-5-nitrophenol (500 mg, 3.25 mmol) in H_2O (6 mL) and DCM (24 mL) was added potassium carbonate (K_2CO_3) (910 mg, 6.49 mmol) at rt. The reaction was then heated to 40 ºC for 2 hours. The reaction was diluted with H_2O and extracted with DCM (3x). The combined organic layers were washed with brine, dried over Na_2SO_4, filtered, and concentrated. The resulting residue was suspended in MeOH (24 mL) followed by the sequential addition of sodium cyanoborohydride ($NaBH_3CN$) (408 mg, 6.49 mmol) and AcOH (0.19 mL, 3.25 mmol) at rt. The reaction was then heated to 65 °C for 2 hr. The reaction was quenched with sat. aq. $NaHCO_3$ and extracted with EtOAc (3x). The combined organic layers were washed with brine, concentrated, and purified via flash chromatography (Teledyne ISCO flash purification system; silica gel column; hexanes : EtOAc; 0-30% EtOAc gradient) to afford the desired product 7-nitro-3-phenyl-3,4-dihydro-*2H*-benzo[*b*][1,4]oxazine (668 mg, 2.61 mmol, 80% yield) as a yellow solid.

^{1}H NMR (400 MHz, DMSO-*d6*): δ 7.99 (d, *J* = 1.9 Hz, 1H), 7.76 (dd, *J* = 8.9, 2.6 Hz, 1H), 7.55 (dd, *J* = 2.6, 0.7 Hz, 1H), 7.43 – 7.32 (m, 5H), 6.79 (d, *J* = 8.9 Hz, 1H), 4.71 – 4.68 (m, 1H), 4.33 – 4.29 (m, 1H), 4.01 (dd, *J* = 10.8, 6.6 Hz, 1H).

^{13}C NMR (100 MHz, DMSO-*d6*): δ 142.4, 140.8, 138.8, 135.9, 128.6, 128.1, 127.1, 119.5, 112.4, 111.4, 69.0, 52.5.

HRMS (ESI): Calculated for formula $C_{14}H_{13}N_2O_3$ ($[M+H]^+$) 257.0921, found 257.0924.

Step 2: Synthesis of 4-methyl-7-nitro-3-phenyl-3,4-dihydro-*2H*-benzo[*b*][1,4]oxazine

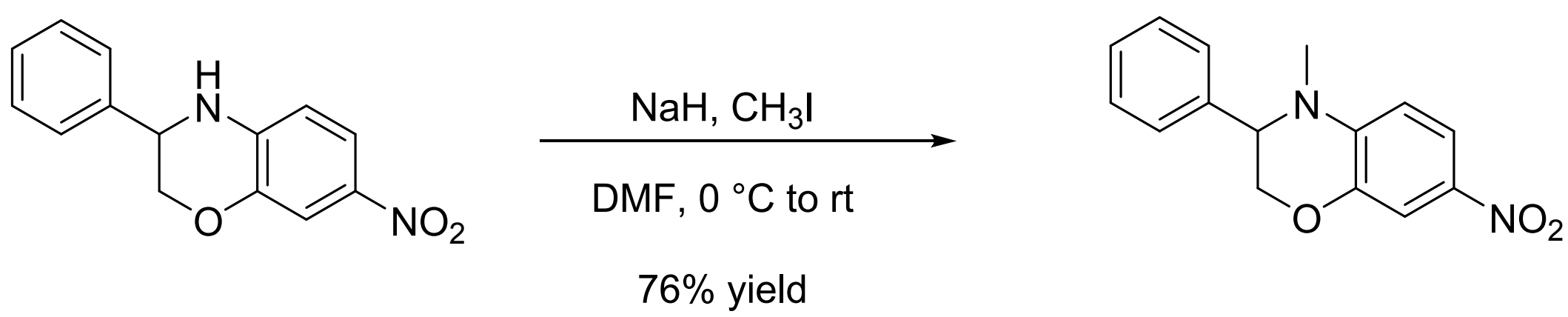

To a solution of 7-nitro-3-phenyl-3,4-dihydro-*2H*-benzo[*b*][1,4]oxazine (668 mg, 2.61 mmol) in DMF (6.5 mL) cooled to 0 °C was added NaH (60% dispersion in mineral oil) (93.8 mg, 3.91 mmol). The reaction was stirred at 0 °C for 5 min followed by the addition of iodomethane (0.32 mL, 5.21 mmol). The reaction was warmed to rt and stirred for 1 hr. The reaction was quenched with the addition of sat. aq. NH_4Cl and extracted with EtOAc. The organic layer was washed with brine, concentrated, and purified via flash chromatography (Teledyne ISCO flash purification system; silica gel column; hexanes : EtOAc; 0-30% EtOAc gradient) to afford the desired product 4-methyl-7-nitro-3-phenyl-3,4-dihydro-*2H*-benzo[*b*][1,4]oxazine (538 mg, 1.99 mmol, 76% yield) as a yellow solid.

^{1}H NMR (400 MHz, DMSO-*d6*): δ 7.87 (dd, *J* = 9.1, 2.6 Hz, 1H), 7.53 (d, *J* = 2.6 Hz, 1H), 7.39 – 7.28 (m, 3H), 7.19 – 7.16 (m, 2H), 6.89 (d, *J* = 9.1 Hz, 1H), 4.80 (t, *J* = 2.9 Hz, 1H), 4.33 – 4.25 (m, 2H), 3.00 (s, 3H).

^{13}C NMR (100 MHz, DMSO-*d6*): δ 142.6, 142.0, 138.9, 135.9, 128.7, 127.8, 126.6, 119.8, 110.5, 109.5, 68.7, 60.2, 36.9.

HRMS (ESI): Calculated for formula $C_{15}H_{15}N_2O_3$ ($[M+H]^+$) 271.1.077, found 271.1082.

Step 3: Synthesis of 4-fluoro-*N*-(4-methyl-3-phenyl-3,4-dihydro-*2H*-benzo[*b*][1,4]oxazin-7-yl)benzamide (7)

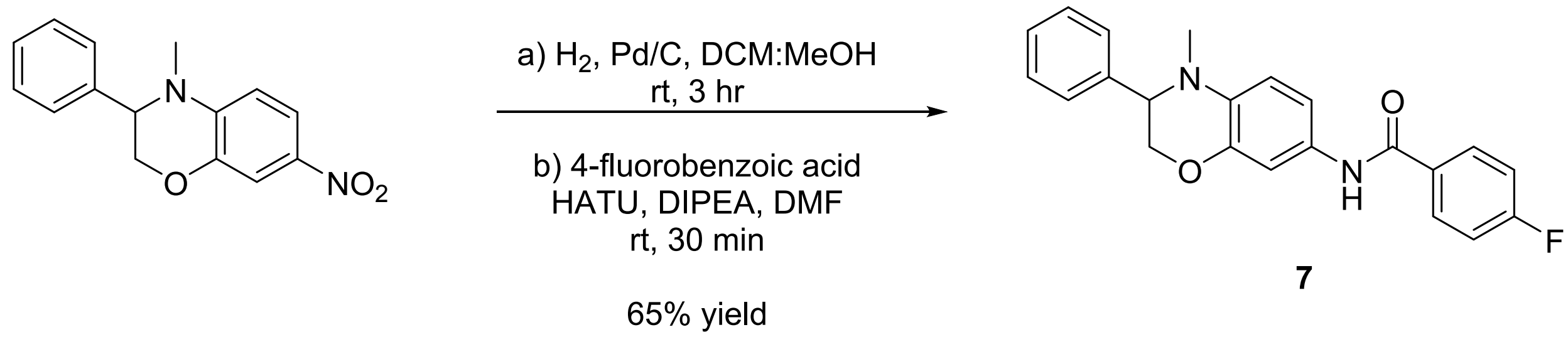

7

To a solution of 4-methyl-7-nitro-3-phenyl-3,4-dihydro-*2H*-benzo[*b*][1,4]oxazine (50 mg, 0.19 mmol) in DCM (0.5 mL) and MeOH (1.0 mL) was added palladium on activated carbon (Pd/C) (10% wt Pd basis) (9.8 mg, 0.009 mmol). The vial was purged via vacuum, and back-filled with H_2 (g). This process was repeated for a total of 3 times. The reaction was stirred at rt for 3 hr under a balloon of H_2. The reaction was diluted with MeOH, filtered, concentrated, and dissolved in DMF (1.0 mL) (henceforth referred to as **solution A**).

In a separate vial, 4-fluorobenzoic acid (31.1 mg, 0.22 mmol) and HATU (84 mg, 0.22 mmol) were dissolved in DMF (1.0 mL) followed by the addition of *N*,*N*-diisopropylethylamine (DIPEA) (64.4 uL, 0.37 mmol). The reaction was stirred at rt for 10 minutes. To this solution was added **solution A** followed by stirring at rt for 30 min. The reaction mixture was purified via reverse phase HPLC (5-95% MeCN/ 0.05% aq. NH_4OH) to afford 4-fluoro-*N*-(4-methyl-3-phenyl-3,4-dihydro-*2H*-benzo[*b*][1,4]oxazin-7-yl)benzamide (**7**) (44 mg, 65% yield) as a beige solid.

^{1}H NMR (400 MHz, DMSO-*d6*): δ 9.99 (s, 1H), 8.02 – 7.98 (m, 2H), 7.38 – 7.23 (m, 9H), 6.78 (d, J = 8.7 Hz, 1H), 4.41 (dd, J = 5.0, 3.0 Hz, 1H), 4.26 (dd, J = 11.0, 3.0 Hz, 1H), 4.16 (dd, J = 10.9, 5.0 Hz, 1H), 2.74 (s, 3H).

^{13}C NMR (100 MHz, DMSO-*d6*): δ 163.9 (d, J = 248.6 Hz), 163.7, 143.3, 139.5, 133.0, 131.6 (d, J = 2.9 Hz), 130.2 (d, J = 9.0 Hz), 129.4, 128.6, 127.6, 127.2, 115.2 (d, J = 21.6 Hz), 114.3, 111.8, 108.7, 69.5, 60.1, 36.5.

HRMS (ESI): Calculated for formula $C_{22}H_{20}N_2O_2$ ($[M+H]^+$) 363.4124, found 363.1511.

Table S1. SAR Table of Select Examples

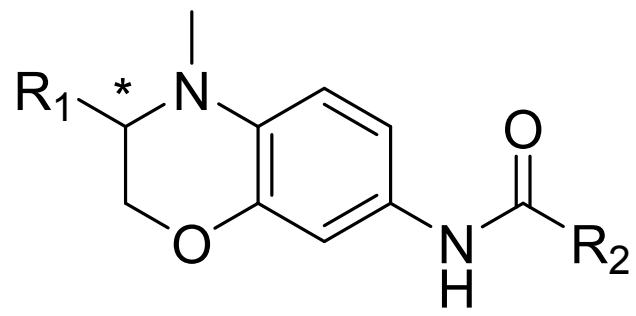

Compound	R_1	*	R_2	h $mGlu_3$ GIRK EC_{50} (nM) [$\%Glu_{max}$]
**7**	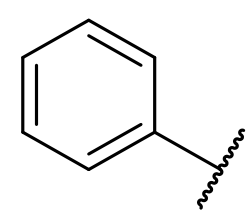	**racemic**	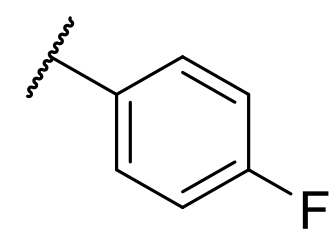	**835 [44%]**
**S1**	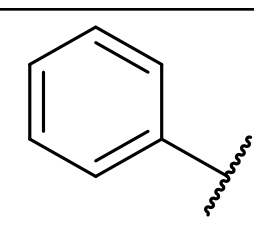	**racemic**	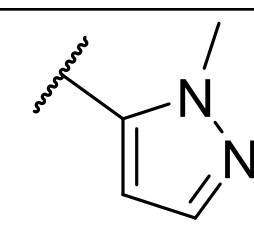	**1157 [57%]**
**S2**	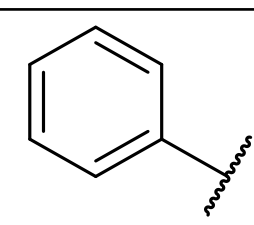	**racemic**	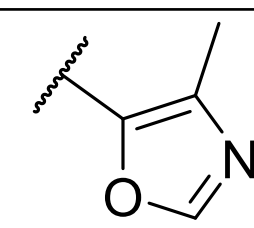	**1150 [81%]**
**S3**	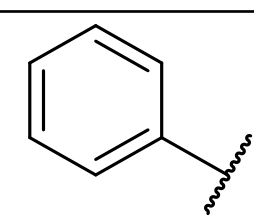	**racemic**	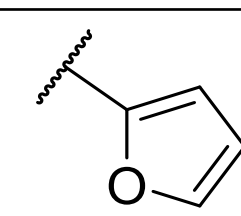	**784 [55%]**
**S4**	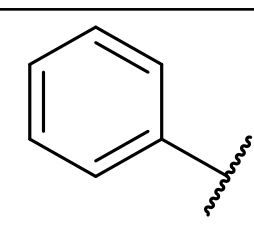	**racemic**	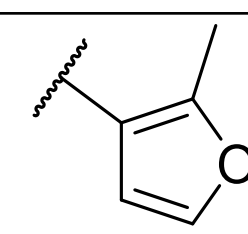	**2107 [66%]**
**S5**	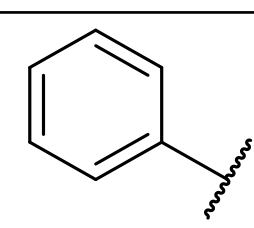	**racemic**	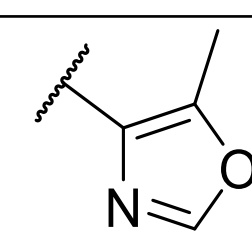	**3823 [75%]**
**S6**	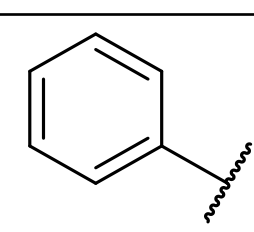	**racemic**	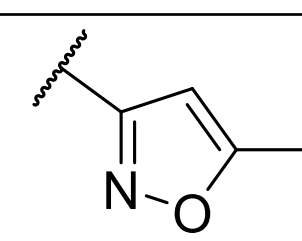	**1783 [64%]**
**S7**	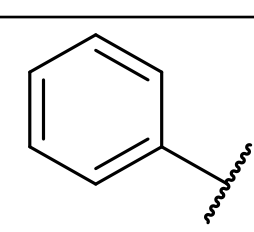	**racemic**	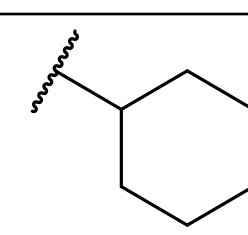	**> 10,000 [70%]**
**S8**	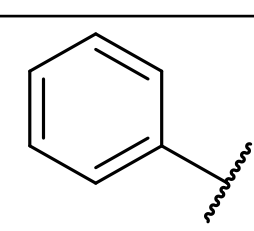	**racemic**	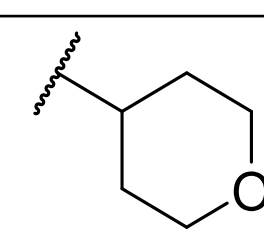	**Inactive**
**S9**	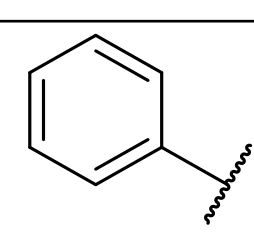	**racemic**	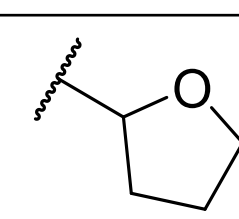	**1182 [58%]**

**S10**	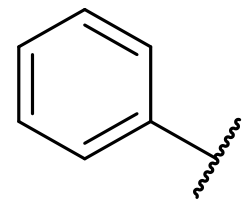	**racemic**	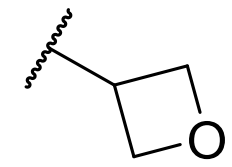	**1590 [57%]**
**S11**	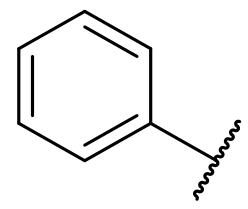	**racemic**	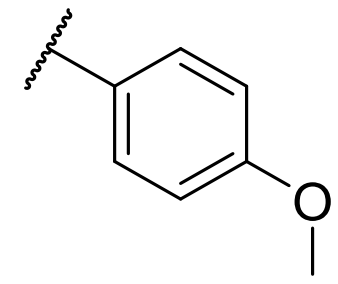	**7100 [77%]**
**S12**	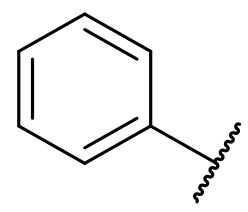	**racemic**	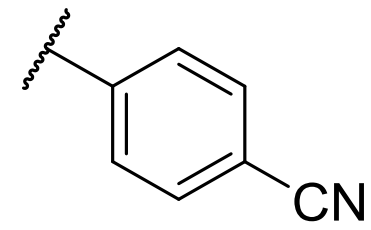	**Inactive**
**S13**	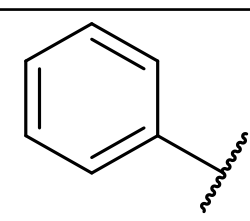	**racemic**	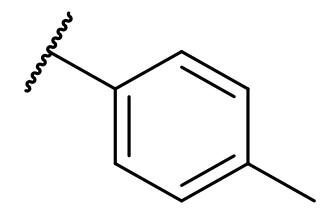	**4995 [51%]**
**S14**	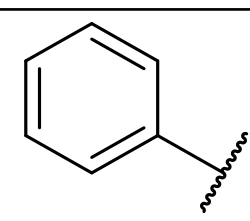	**racemic**	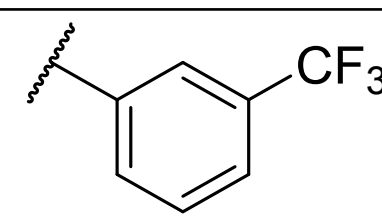	**Inactive**
**S15**	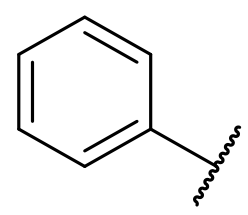	**racemic**	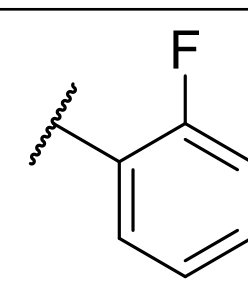	**1530 [54%]**
**S16**	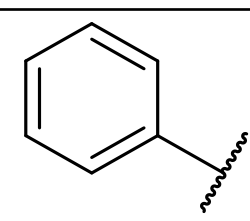	**racemic**	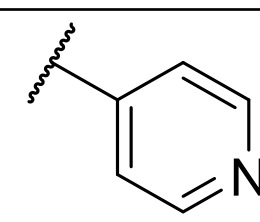	**616 [54%]**
**S17**	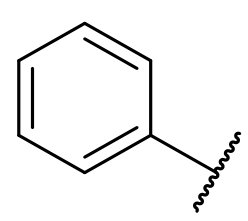	**racemic**	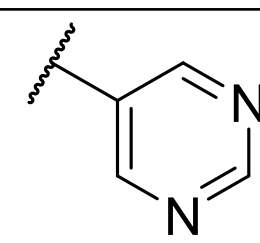	**1486 [70%]**
**S18**	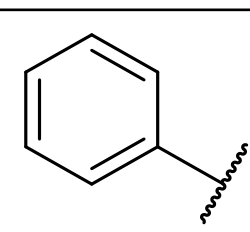	**racemic**	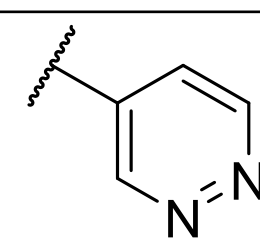	**908 [61%]**
**S19**	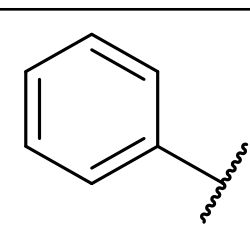	**racemic**	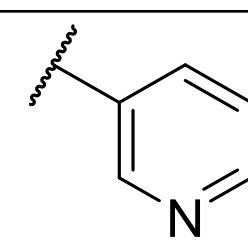	**447 [67%]**
**S20**	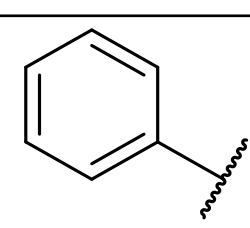	**racemic**	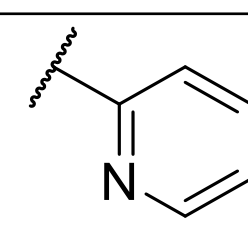	**3087 [67%]**
**S21**	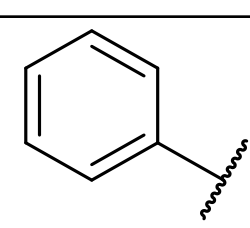	**racemic**	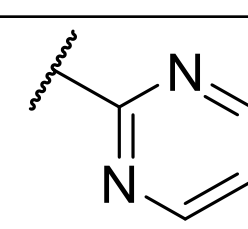	**279 [78%]**
**S22**	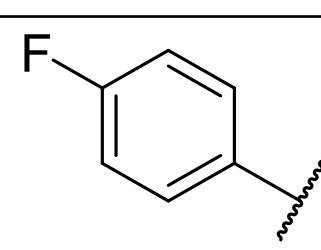	**racemic**	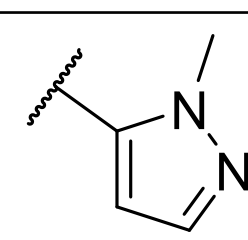	**439 [39%]**
**S23**	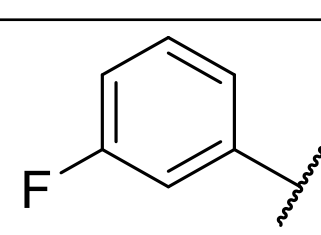	**racemic**	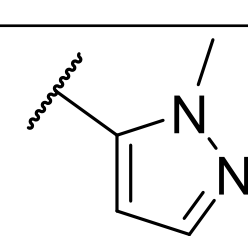	**687 [51%]**

**S24**	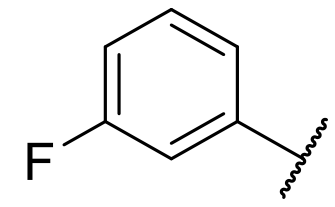	**racemic**	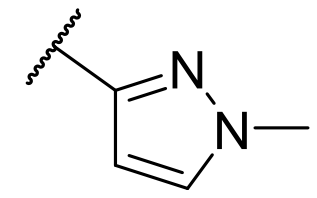	**1100 [63%]**
**S25**	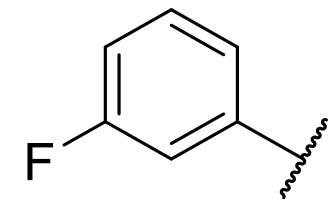	**racemic**	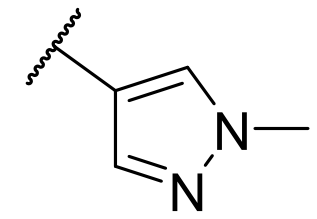	**138 [57%]**
**S26**	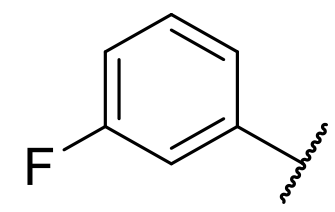	**racemic**	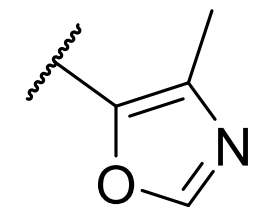	**1120 [58%]**
**S27**	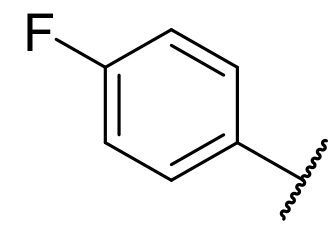	**racemic**	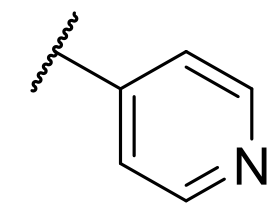	**Inactive**
**S28**	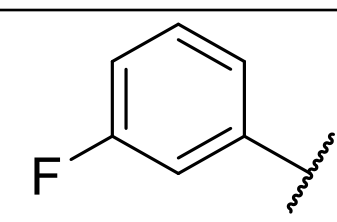	**racemic**	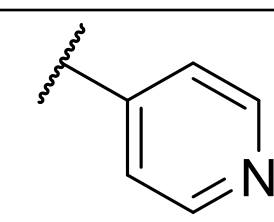	**285 [46%]**
**S29**	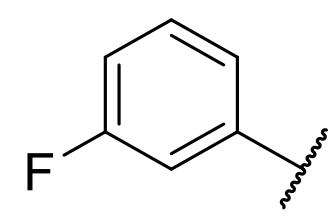	**racemic**	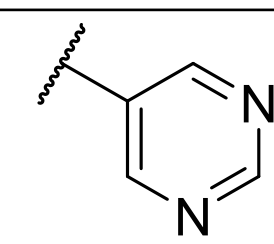	**301 [55%]**
**S30**	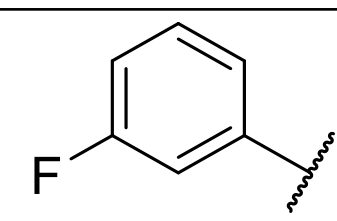	**racemic**	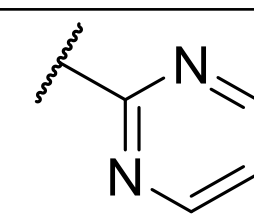	**180 [74%]**
**8**	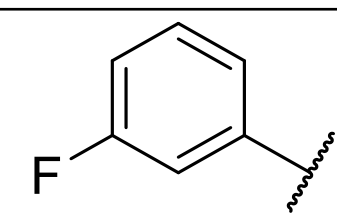	**(*S*)**	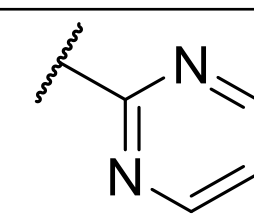	**36 [79%]**
**9**	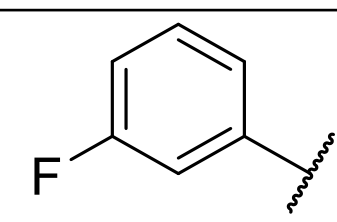	**(*R*)**	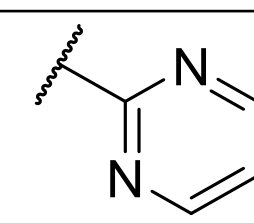	**2500 [52%]**

Chiral SFC Separation for 8 and 9

Racemic Intermediate A was prepared according to the method described previously (for compound **7**) using appropriate starting materials.

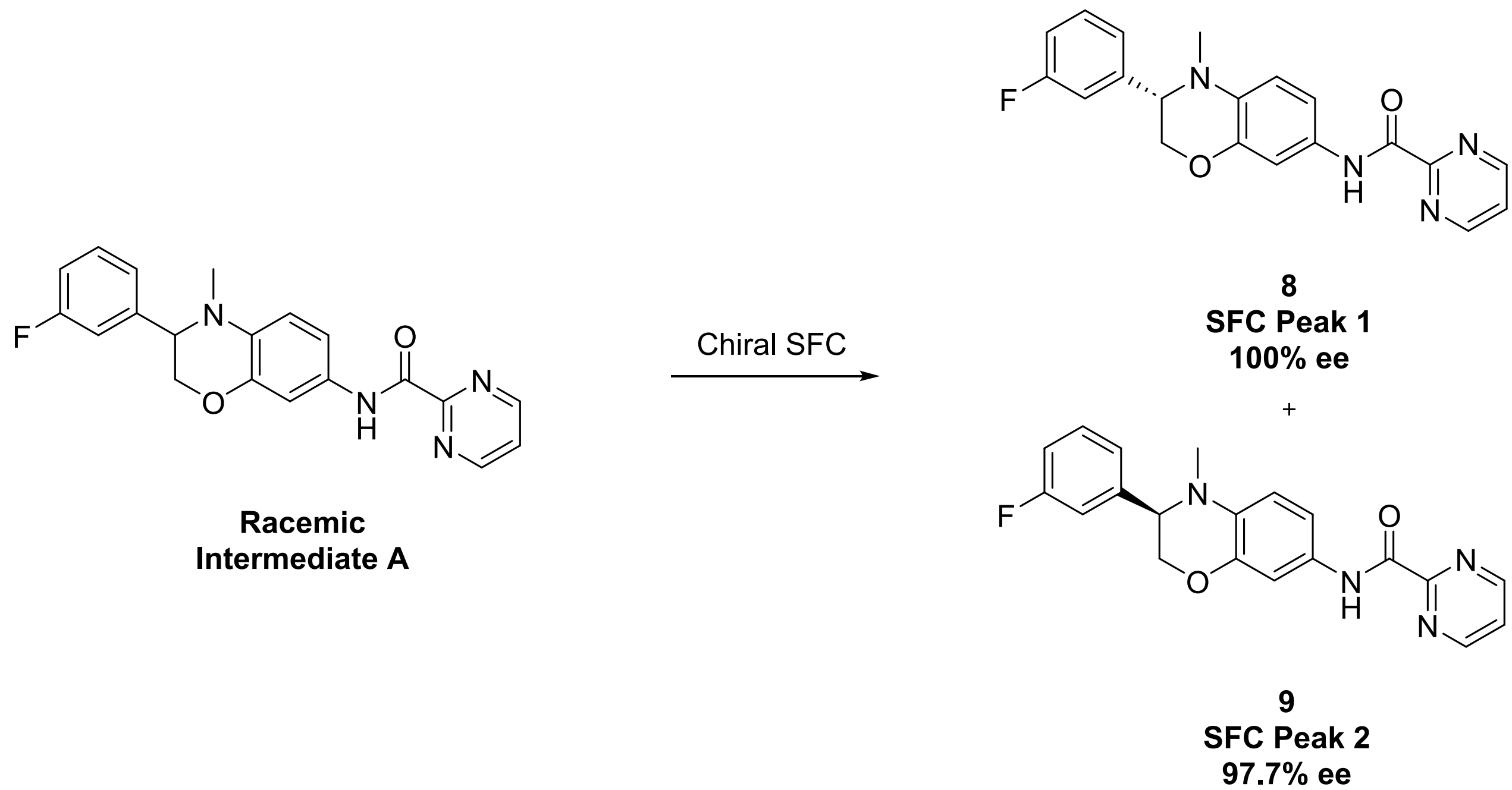

Analytical Conditions
Column: Chiralpak IA; Column Dimensions: 4.6 x 250 mm; Particle Size: 5 micron; Co-solvent: Methanol; Modifier: 0.1% DEA; Gradient Profile: 50% Isocratic; Flow Rate (mL/min): 3.5; Backpressure (bar): 100; Column Temperature: 40 °C

Preparative Conditions
Column: Chiralpak IA; Column Dimensions: 21 x 250 mm; Particle Size: 5 micron; Co-solvent: Methanol; Modifier: None; Gradient Profile: 50% Isocratic; Flow Rate (mL/min): 80; Backpressure (bar): 100; Column Temperature: 40 ˚C

The absolute configuration of **8** was confirmed via a chiral synthetic approach (page S10) with confirmation of pharmacological potency data of **8** prepared by both the chiral synthesis and SFC separation.

Chiral SFC Chromatogram for Racemic Intermediate A

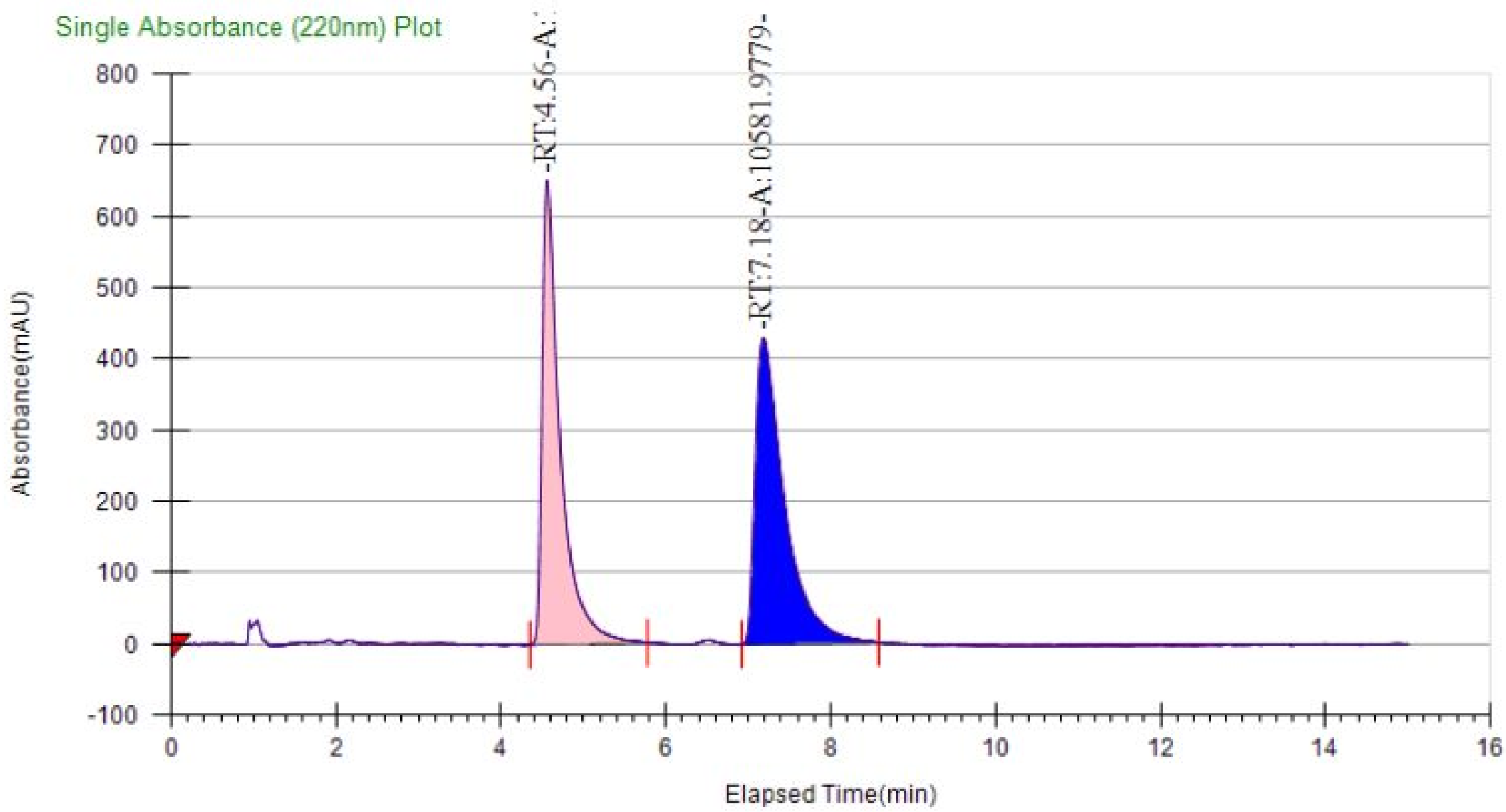

General Information

Log Author	Log Date	Report By	Report Date	Notes
Administrator	9/30/2021 11:34:00 AM	Administrator	9/30/2021	

Run Information

Instrument Method	Inj. Vol.	Solvent	Column	Sample	Well Location	Temp.	Flow	% Modifier	Pressure
50 Isocratic	10	1: MeOH (0.1%DEA)	IA(4.6 x 250mm)	CWR-22-135 Racemic	12F	40	3.5	50	100

Peak Information

Peak No	% Area	Area	Ret. Time	Height	Cap. Factor
1	48.6194	10013.2888	4.56 min	650.2438	4558.95
2	51.3806	10581.9779	7.18 min	428.7398	7182.1

Chiral SFC Chromatogram for SFC Peak 1 (8)

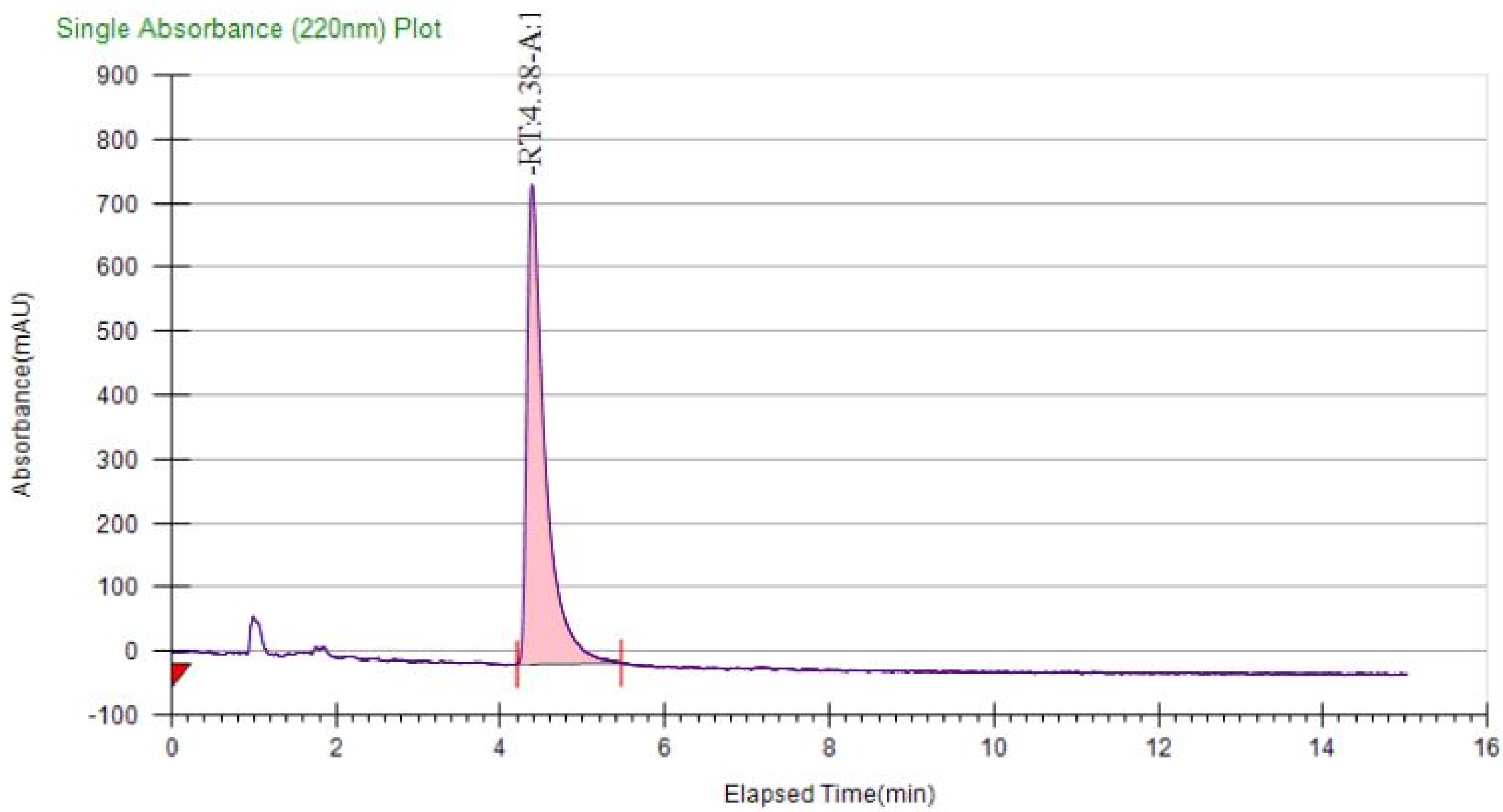

General Information

Log Author	Log Date	Report By	Report Date	Notes
Administrator	9/30/2021 9:52:38 AM	Administrator	9/30/2021	

Run Information

Instrument Method	Inj. Vol.	Solvent	Column	Sample	Well Location	Temp.	Flow	% Modifier	Pressure
50 Isocratic	10	1: MeOH (0.1%DEA)	IA(4.6 x 250mm)	CWR-22-135 Peak 1	13D	40	3.5	50	100

Peak Information

Peak No	% Area	Area	Ret. Time	Height	Cap. Factor
1	100	11392.2498	4.38 min	749.0566	0

Chiral SFC Chromatogram for SFC Peak 2 (9)

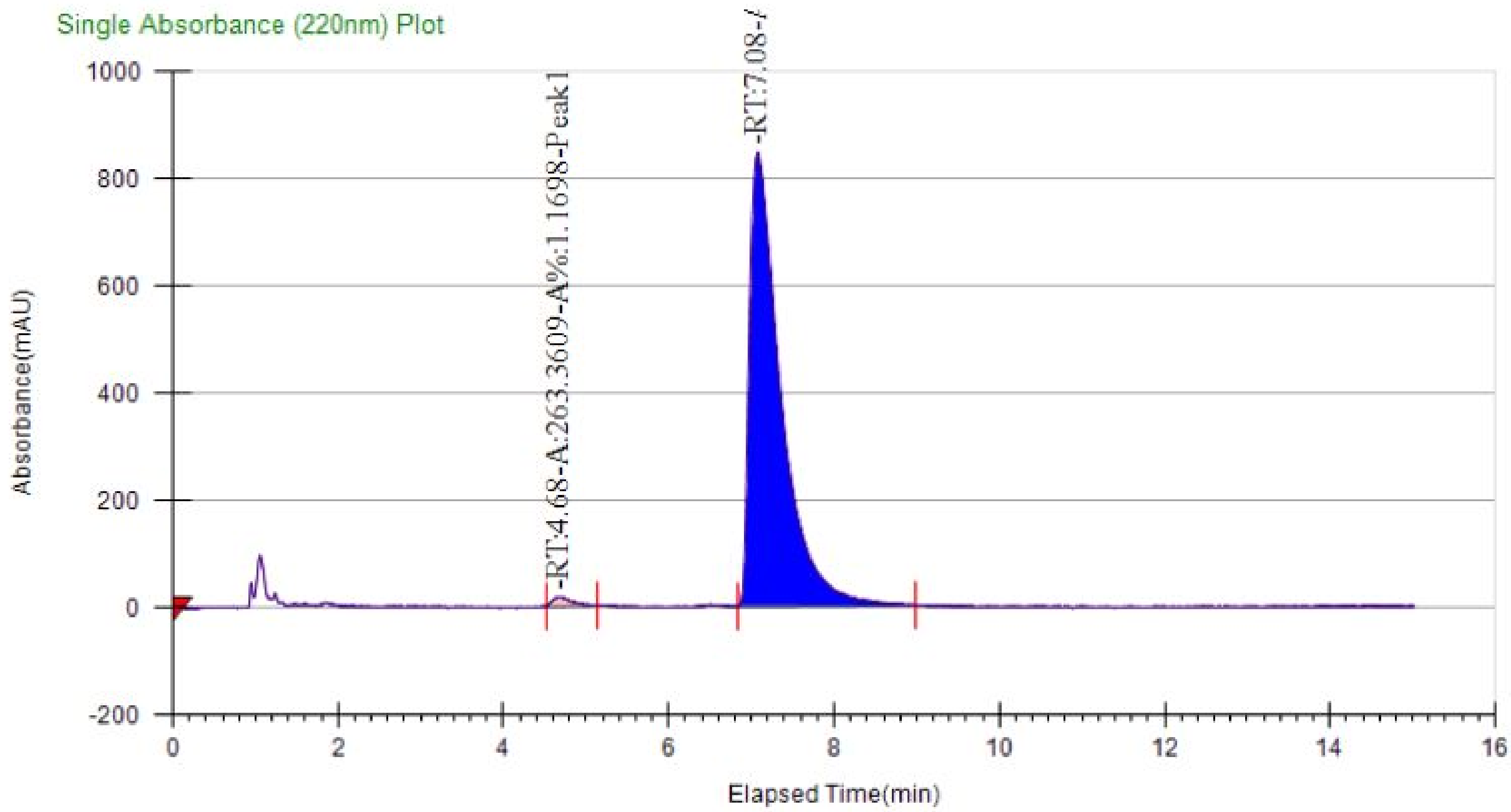

General Information

Log Author	Log Date	Report By	Report Date	Notes
Administrator	9/30/2021 11:09:18 AM	Administrator	9/30/2021	

Run Information

Instrument Method	Inj. Vol.	Solvent	Column	Sample	Well Location	Temp.	Flow	% Modifier	Pressure
50 Isocratic	10	1: MeOH (0.1%DEA)	IA(4.6 x 250mm)	CWR-22-135 Peak 2	13E	40	3.5	50	100

Peak Information

Peak No	% Area	Area	Ret. Time	Height	Cap. Factor
1	1.1698	263.3609	4.68 min	17.139	4678.95
2	98.8302	22250.5959	7.08 min	846.8709	7078.7667

Chiral Synthesis of VU6052959 (8)

Step 1: Synthesis of *tert*-butyl (*S*)-(2-(2-bromo-5-nitrophenoxy)-1-(3-fluorophenyl)ethyl)carbamate (13)

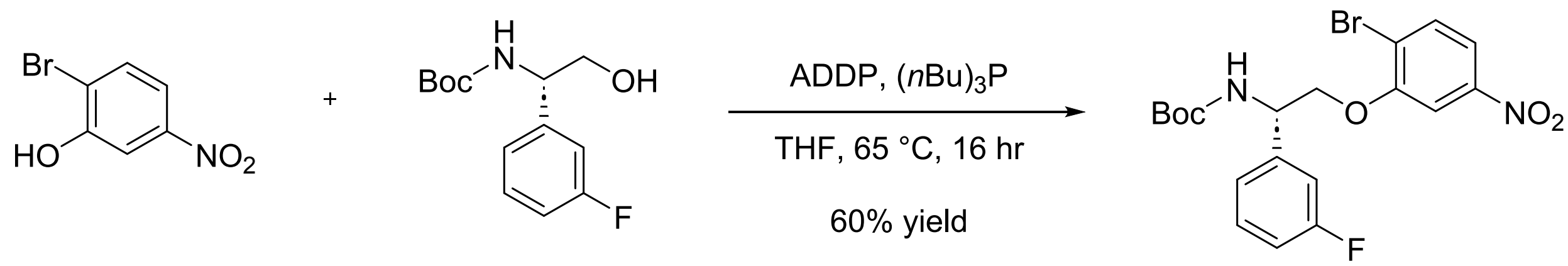

12 **11** **13**

To a solution of 2-bromo-5-nitrophenol (**12**) (19.6 g, 89.9 mmol) and *tert*-butyl (*S*)-(1-(3-fluorophenyl)-2-hydroxyethyl)carbamate (**11**) (27.5 g, 107.9 mmol) in THF (300 mL) was sequentially added 1,1'-(azodicarbonyl)dipiperidine (ADDP) (34.0 g, 134.9 mmol) and tri-*N*-butylphosphine ((*n*Bu)$_3$P) (33.7 mL, 134.9 mmol) dropwise at rt. Upon completion of addition, the reaction was heated to 75 °C and for 3 hr. The reaction was recharged with additional ADDP (34.0 g, 134.9 mmol) and (*n*Bu)$_3$P (33.7 mL, 134.9 mmol), and the resulting reaction was heated to 75 °C for 16 hr. Upon cooling to rt, the reaction was quenched with sat. aq. $NaHCO_3$ and extracted with EtOAc (3x). The combined organic material was concentrated and purified via flash chromatography (Teledyne ISCO flash purification system; silica gel column; hexanes : EtOAc; 0-30% EtOAc gradient) to afford *tert*-butyl (*S*)-(2-(2-bromo-5-nitrophenoxy)-1-(3-fluorophenyl)ethyl)carbamate (**13**) (24.6 g, 54.0 mmol, 60% yield) as a light orange solid.

^{1}H NMR (400 MHz, DMSO-*d6*): δ 7.91 – 7.85 (m, 2H), 7.76 (dd, *J* = 8.7, 2.5 Hz, 1H), 7.64 (d, *J* = 8.7 Hz, 1H), 7.44 – 7.38 (m, 1H), 7.32 – 7.27 (m, 2H), 7.15 – 7.09 (m, 1H), 5.09 – 5.03 (m, 1H), 4.41 – 4.36 (m, 1H), 4.30 – 4.24 (m, 1H), 1.39 (s, 9H).

^{13}C NMR (100 MHz, DMSO-*d6*): δ 162.2 (d, *J* = 243.2 Hz), 155.1, 155.0, 147.9, 142.7 (d, *J* = 7.1 Hz), 133.8, 130.3 (d, *J* = 8.1 Hz), 123.3 (d, *J* = 2.8 Hz), 119.1, 116.9, 114.2 (d, *J* = 20.9 Hz), 114.0 (d, *J* = 22.1 Hz), 108.3, 78.2, 71.5, 53.0, 28.2.

HRMS (ESI): Calculated for formula $C_{19}H_{19}BrFN_2O_5Na$ ([M+Na]$^+$) 477.0432, found 477.0436.

Step 2: Synthesis of *tert*-butyl (*S*)-3-(3-fluorophenyl)-7-nitro-2,3-dihydro-4*H*-benzo[*b*][1,4]oxazine-4-carboxylate (14)

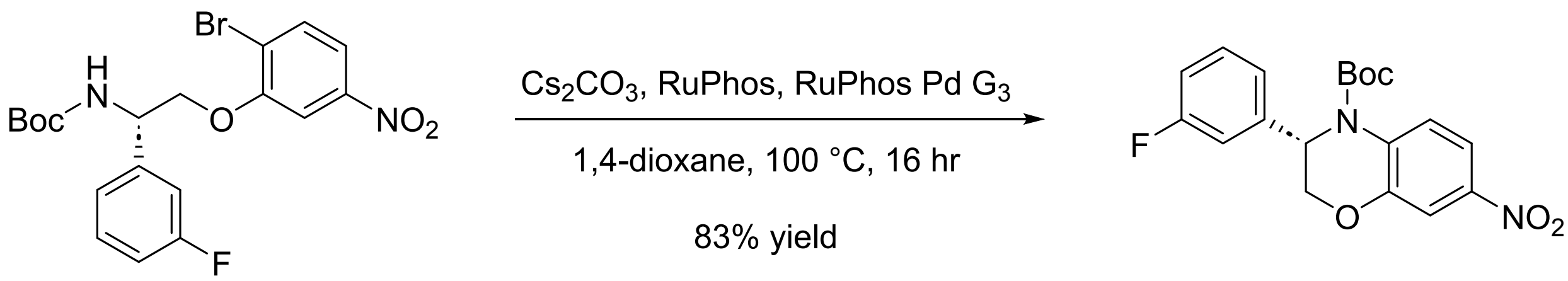

13 **14**

To a suspension of *tert*-butyl (*S*)-(2-(2-bromo-5-nitrophenoxy)-1-(3-fluorophenyl)ethyl)carbamate (**13**) (24.6 g, 54.0 mmol) and Cs_2CO_3 (42.5 g, 129.7 mmol) in 1,4-dioxane (1.1 L) was added RuPhos (1.3 g, 2.7 mmol) and RuPhos Pd G_3 (2.3 g, 2.7 mmol). The resulting mixture was sparged with N_2 for 15 min before the reaction was heated to 100 °C for 3 hr. The reaction was recharged with additional RuPhos (1.3 g, 2.7 mmol) and RuPhos Pd G_3 (2.3 g, 2.7 mmol), and the reaction was heated to 100 °C for an additional 3 hr. Upon cooling to rt, the reaction mixture was diluted with EtOAc, filtered through Celite, concentrated, and purified via flash chromatography (Teledyne ISCO flash purification system; silica gel column; hexanes : EtOAc; 0-20% EtOAc gradient) to afford *tert*-butyl (*S*)-3-(3-fluorophenyl)-7-nitro-2,3-dihydro-4*H*-benzo[*b*][1,4]oxazine-4-carboxylate (**14**) (16.7 g, 44.6 mmol, 83% yield) as a yellow foam.

^{1}H NMR (400 MHz, DMSO-*d6*): δ 8.35 (d, *J* = 9.4 Hz, 1H), 7.86 (dd, *J* = 9.3, 2.8 Hz, 1H), 7.66 (d, *J* = 2.8 Hz, 1H), 7.40 – 7.34 (m, 1H), 7.13 – 7.07 (m, 2H), 7.04 – 7.01 (m, 1H), 5.73 – 5.71 (m, 1H), 4.75 (dd, *J* = 11.7, 1.9 Hz, 1H), 4.41 (dd, *J* = 11.7, 2.9 Hz, 1H), 1.43 (s, 9H).

^{13}C NMR (100 MHz, DMSO- *d6*): δ 162.2 (d, *J* = 243.8 Hz), 151.4, 145.1, 142.2, 141.4 (d, *J* = 7.0 Hz), 132.4, 130.7 (d, *J* = 8.2 Hz), 122.1, 122.0 (d, *J* = 2.7 Hz), 116.6, 114.4 (d, *J* = 20.9 Hz), 113.1 (d, *J* = 22.5 Hz), 112.0, 82.8, 67.9, 54.2, 27.6.

HRMS (ESI): Calculated for formula $C_{19}H_{20}FN_2O_5$ ($[M+H]^+$) 375.1351, found 375.1347.

Step 3: Synthesis of (*S*)-3-(3-fluorophenyl)-4-methyl-7-nitro-3,4-dihydro-2*H*-benzo[*b*][1,4]oxazine (15)

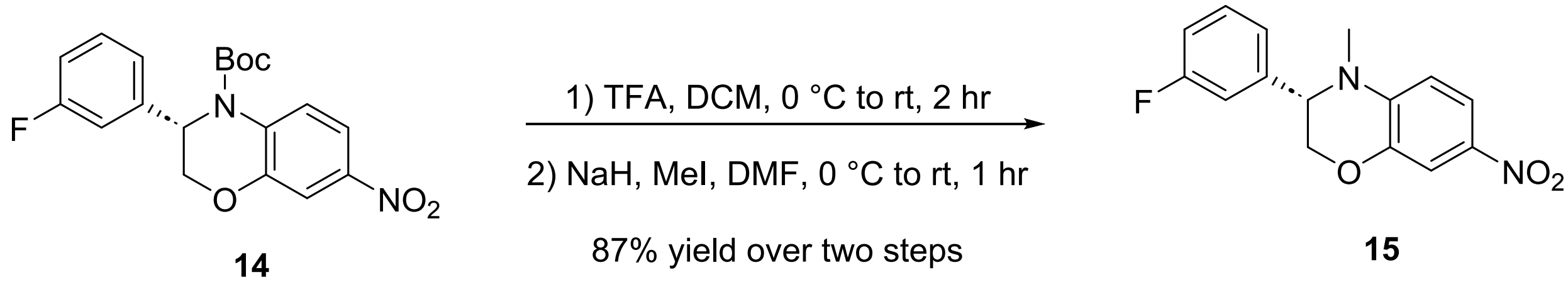

To a solution of *tert*-butyl (*S*)-3-(3-fluorophenyl)-7-nitro-2,3-dihydro-4*H*-benzo[*b*][1,4]oxazine-4-carboxylate (**14**) (16.7 g, 44.6 mmol) in DCM (75 mL) cooled to 0 °C was added TFA (34.2 mL, 446.1 mmol), and the reaction was allowed to warm to rt and stir for 1.5 hr. The reaction mixture was concentrated, and the resulting residue was basified with sat. aq. $NaHCO_3$ and extracted with EtOAc (3x). The combined organic material was concentrated and dissolved in DMF (150 mL). The reaction was cooled to 0 °C before the portionwise addition of NaH (60% dispersion in mineral oil) (2.7 g, 66.9 mmol). The reaction was then stirred at 0 °C for 10 min before the addition of iodomethane (5.6 mL, 89.2 mmol). Upon completion of addition, the reaction was warmed to rt and stirred for 1 hr. The reaction was quenched with sat. aq. NH_4Cl and extracted with EtOAc. The organic layer was then washed with brine, concentrated, and purified via flash chromatography (Teledyne ISCO flash purification system; silica gel column; hexanes : EtOAc; 0-40% EtOAc gradient) to afford (*S*)-3-(3-fluorophenyl)-4-methyl-7-nitro-3,4-dihydro-2*H*-benzo[*b*][1,4]oxazine (**15**) (11.2 g, 38.9 mmol, 87% yield) as a yellow solid.

^{1}H NMR (400 MHz, DMSO-*d6*): δ 7.87 (dd, J = 9.1, 2.7 Hz, 1H), 7.54 (d, J = 2.7 Hz, 1H), 7.44 – 7.38 (m, 1H), 7.17 – 7.12 (m, 1H), 7.04 – 6.99 (m, 2H), 6.90 (d, J = 9.1 Hz, 1H), 4.85 (t, J = 2.8 Hz, 1H), 4.36 (dd, J = 11.3, 2.6 Hz, 1H), 4.27 (dd, J = 11.3, 3.0 Hz, 1H), 3.02 (s, 3H).

^{13}C NMR (100 MHz, DMSO-*d6*): δ 162.3 (d, J = 244.2 Hz), 142.2, 142.1 (d, J = 6.7 Hz), 141.9, 136.1, 130.8 (d, J = 8.3 Hz), 122.6 (d, J = 2.8 Hz), 119.8, 114.6 (d, J = 20.9 Hz), 113.6 (d, J = 21.9 Hz), 110.6, 109.7, 68.4, 59.8 (d, J = 1.9 Hz), 37.0.

HRMS (ESI): Calculated for formula $C_{15}H_{14}FN_2O_3$ ($[M+H]^+$) 289.0983, found 289.0992.

Step 4: Synthesis of (*S*)-*N*-(3-(3-fluorophenyl)-4-methyl-3,4-dihydro-2*H*-benzo[*b*][1,4]oxazin-7-yl)pyrimidine-2-carboxamide (8)

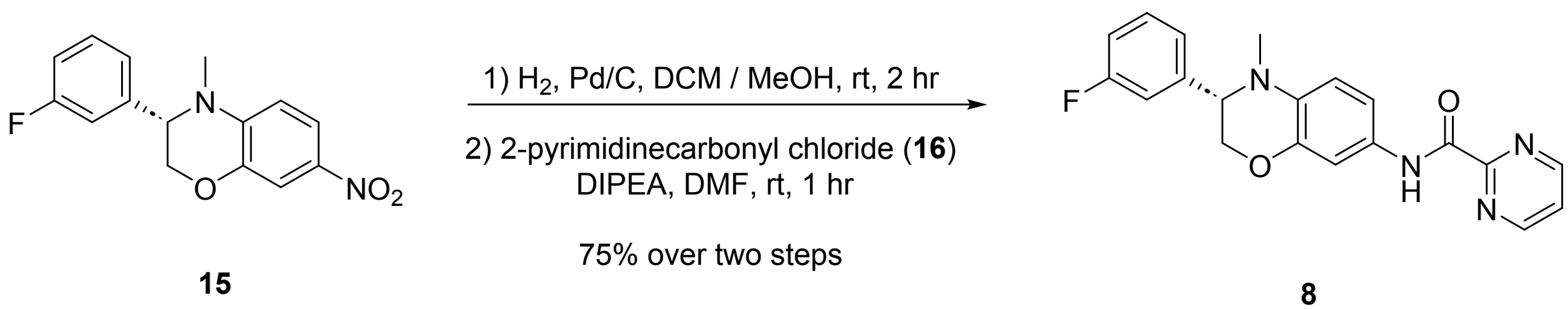

To a solution of (*S*)-3-(3-fluorophenyl)-4-methyl-7-nitro-3,4-dihydro-2*H*-benzo[*b*][1,4]oxazine (**15**) (7.0 g, 24.3 mmol) in DCM (140 mL) and MeOH (40 mL) was added Pd/C (10% wt Pd basis) (2.6 g, 2.4 mmol). The flask was purged via vacuum, and back-filled with H_2 (g). This process was repeated for a total of 3 times. The reaction was stirred at rt for 2 hr under a balloon of H_2. The reaction was diluted with EtOAc, filtered through Celite, and concentrated. The residue was dissolved in DMF (120 mL) followed by the addition of DIPEA (30 mL, 172.2 mmol). Pyrimidine-2-carbonyl chloride (**16**) (10.4 g, 72.9 mmol) (added in 4 portions over a total time of 20 min) was added at rt. The reaction mixture was stirred at rt for 1 hr. The reaction was diluted with EtOAc and quenched with sat. aq. $NaHCO_3$ and H_2O. The layers were separated, and the organic layer was washed with H_2O (2x) and brine. The organic layer was then concentrated and purified via flash chromatography (Teledyne ISCO flash purification system; silica gel column; hexane : EtOAc; 0-100% EtOAc gradient) to afford (*S*)-*N*-(3-(3-fluorophenyl)-4-methyl-3,4-dihydro-2*H*-benzo[*b*][1,4]oxazin-7-yl)pyrimidine-2-carboxamide (**8**) (6.7 g, 18.3 mmol, 75% yield) as a yellow solid.

^{1}H NMR (400 MHz, DMSO-*d6*): δ 10.51 (s, 1H), 9.01 (d, J = 4.9 Hz, 2H), 7.71 (t, J = 4.9 Hz, 1H), 7.43 – 7.36 (m, 3H), 7.15 – 7.03 (m, 3H), 6.79 (d, J = 8.7 Hz, 1H), 4.50 (t, J = 3.6 Hz, 1H), 4.26 (dd, J = 11.0, 3.0 Hz, 1H), 4.20 (dd, J = 11.0, 4.3 Hz, 1H), 2.78 (s, 3H).

^{13}C NMR (100 MHz, DMSO-*d6*): δ 162.3 (d, J = 243.7 Hz), 160.3, 158.5, 157.8, 143.1, 142.7 (d, J = 6.5 Hz), 132.9, 130.6 (d, J = 8.3 Hz), 128.8, 123.2 (d, J = 2.8 Hz), 123.0, 114.3 (d, J = 20.7 Hz), 114.3, 113.9 (d, J = 21.5 Hz), 111.8, 108.5, 69.0, 59.6 (d, J = 1.8 Hz), 36.6.

HRMS (ESI): Calculated for formula $C_{20}H_{18}FN_4O_2$ ($[M+H]^+$) 365.1408, found 365.1412.

Table S2. Eurofins Lead Profiling Screen Data

This is a radioligand binding panel of 67 targets including GPCRs, ion channels, transporters and nuclear hormones. Biochemical assay results are presented as the percent inhibition of specific binding at a 10 μM concentration of VU6052959 (**8**). Results showing an inhibition or stimulation higher than 50% are considered to represent significant effect and are highlighted in the table below.

**Target / Protein**	**Species**	**% Inhibition at 10 μM**
Adenosine A_1	human	-2
Adenosine A_{2A}	human	-7
Adenosine A_3	human	31
Adrenergic α_{1A}	human	3
Adrenergic α_{1B}	human	12
Adrenergic α_{1D}	human	20
Adrenergic α_{2A}	human	9
Adrenergic β_1	human	-2
Adrenergic β_2	human	-2
Androgen (testosterone)	human	24
Bradykinin B_1	human	10
Bradykinin B_2	human	20
Calcium Channel L-Type, Benzothiazepine	rat	19
Calcium Channel L-Type, Dihydropyridine	rat	37
Calcium Channel N-Type	rat	16
Cannabinoid CB_1	human	28
Dopamine D_1	human	3
Dopamine D_{2S}	human	8
Dopamine D_3	human	11
Dopamine $D_{4.4}$	human	5
Endothelin ET_A	human	-12
Endothelin ET_B	human	6
Epidermal Growth Factor (EGF)	human	-8
Estrogen ERα	human	6
$GABA_A$, Flunitrazepam, Central	rat	6
$GABA_A$, Muscimol, Central	rat	16
$GABA_{B1A}$	human	12
Glucocorticoid	human	19
Glutamate, Kainate	rat	10
Glutamate, NMDA, Agonism	rat	12
Glutamate, NMDA, Glycine	rat	-3

Glutamate, NMDA, Phencyclidine	rat	-2
Histamine H_1	human	11
Histamine H_2	human	11
Histamine H_3	human	37
Imidazoline I_2, Central	rat	-18
Interleukin IL-1 R1	human	-11
Leukotriene, Cysteinyl $CysLT_1$	human	5
Melatonin MT_1	human	1
Muscarinic M_1	human	0
Muscarinic M_2	human	-6
Muscarinic M_3	human	6
Neuropeptide Y Y_1	human	11
Neuropeptide Y Y_2	human	-12
Nicotinic Acetylcholine α1, Bungarotoxin	human	4
Nicotinic Acetylcholine α3β4	human	-2
Opiate δ1 (OP1, DOP)	human	18
Opiate κ (OP2, KOP)	human	11
Opiate μ (OP3, MOP)	human	10
Phorbol Ester	mouse	3
Platelet Activating Factor (PAF)	human	-9
Potassium Channel [K_{ATP}]	hamster	-8
Potassium Channel hERG	human	5
Prostanoid EP_4	human	26
Purinergic P2X	rat	8
Purinergic P2Y, Non-Selective	rat	10
Rolipram	rat	6
Serotonin (5-Hydroxytryptamine) $5\text{-}HT_{1A}$	human	5
Serotonin (5-Hydroxytryptamine) $5\text{-}HT_{2B}$	human	36
Serotonin (5-Hydroxytryptamine) $5\text{-}HT_3$	human	-3
Sigma σ_1	human	-7
Sodium Channel, Site 2	rat	30
Thyroid Hormone	rat	-3
Transporter, Dopamine (DAT)	human	63
Transporter, GABA	rat	-14
Transporter, Norepinephrine (NET)	human	66
Transporter, Serotonin (5-Hydroxytryptamine) (SERT)	human	1

For DAT inhibition, a follow-up functional assay was performed at Eurofins. DAT, IC_{50} = 3.9 μM and K_i = 3.1 μM.

In vitro molecular pharmacology methods (cAMP)

Description of $mGlu_3$ Potentiator Screen (cAMP)

The activation of the Gi-coupled $mGlu_3$ causes an inhibition of intracellular cAMP after stimulation with Forskolin, which can be quantified by use of a suitable assay kit and an adequate plate reader. This technique is used to characterize pharmacological effects of the $mGlu_3$ potentiators by the use of an appropriate cell line.

Materials:

Barcode labelled CELLCOAT® 384 Poly-D-Lysine coated white microplates with lid (Corning)

Nutrient Mixture F12 Ham's w/o Glutamine (Sigma)

1 M HEPES (Sigma)

Forskolin (Sigma)

IBMX (Sigma)

L-Glutamic acid (Sigma)

FBS (GIBCO-Invitrogen)

HBSS 10X with Ca/Mg (GIBCO-Invitrogen)

BSA Fraction V (Protease free) (Serva)

cAMP Gi Kit (Cisbio Bioassays and lysis buffer (Cisbio Bioassays).

Cell line:

The "CHO K1 hGRM cell line" which is the CHO K1 cell line overexpressing the human metabotropic Glutamate 3 receptor (Entrez reference gene ID: 2931; NP_000831.2; NM_000840.2) was purchased from DiscovereX (cAMP Hunter CHO-K1 GRM3 Cell Line; CatNo. 95-0137C2). The CHO K1 hGRM cell line was expanded to larger cell numbers and

the cells were obtained frozen in cryo vials with 1-20 million cells/vial and stored until use at -150°C. The viability of the cells after thawing was >90%.

Method:

The CHO K1 hGRM3 cells are thawed at 37°C and immediately diluted with cell culture medium. After centrifugation (5 minutes at 900 rpm; Heraeus Multifuge X1R), the cell pellet is re-suspended in medium and then distributed from a stirred spinner flask into the wells of columns 1 to 23 of the assay plates (5.000 cells/well; 25 µL/well). The wells of column 24 are left empty to add cAMP for a standard curve later on in the course of the assay. The plates are incubated for one hour at room temperature before they are incubated for 48 hours at 37°C/5% CO_2. After washing the cells in the plate three times with 80 µL HBSS/HEPES buffer (10 µL buffer remaining in the wells after washing), 5 µL per well of compounds diluted in HBSS/HEPES buffer containing 0.2% BSA (final concentration: 0.1%) and 1 mM IBMX (final concentration: 0.5 mM) are added to the wells in columns 1 to 22 of the assay plate. The plates are incubated for 15 minutes at room temperature. Thereafter 5 µL per well of L-Glutamic acid (final concentration: 50-100 nM), forskolin (final concentration: 0.03 µM) and 1 mM IBMX (final concentration: 0.5 mM) dissolved in HBSS/HEPES buffer containing 0.2% BSA (final concentration: 0.1%) are added to the assay plate (final DMSO concentration: 1%). The wells of columns 23 and 24 are used either for the positive and the negative controls or for the cAMP standard curve (for details see below). The assay plate is incubated for 30 minutes at room temperature. Then 5 µL per well of Anti-cAMP-Antibody-d2 solution and 5 µL per well of cAMP-Europium Cryptate dilution are added to all wells of the plate and the plate is incubated another 60 minutes light protected at room temperature. The emissions at 615 nm and 665 nm (Excitation wavelength: 320 nm) are measured on the EnVision™ reader (PerkinElmer). The

ratio between the emission at 665 nm and 615 is calculated by the reader.

Cell culture medium:

Nutrient Mixture F12 Ham's w/o Glutamine; 10% FBS

HBSS/Hepes buffer:

HBSS1x, 20 mM HEPES pH 7.4

Anti-cAMP-Antibody-d2 (cAMP Gi kit):

Prepared by 1:50 dilution of stock with lysis buffer (cAMP Gi kit)

cAMP-Europium Cryptate (cAMP Gi kit):

Prepared by 1:50 dilution of stock with lysis buffer (cAMP Gi kit)

cAMP standard:

The cAMP standard is prepared by diluting the cAMP stock solution with HBSS/Hepes buffer: 5 µL/well of the cAMP dilutions (in HBSS/Hepes buffer containing 1 mM IBMX and 0.2% BSA - final concentration: 0.5 mM IBMX and 0.1% BSA) are added to 10 µL/well HBSS/Hepes buffer plus 5 µL/well 4% DMSO in HBSS/Hepes containing 0.2% BSA (final DMSO concentration: 1%) in the wells of column 24 of the assay plate. The final cAMP concentrations in the assay plate are: 0, 0.17, 0.69, 2.78, 11.1, 44.5, 178, and 712 nM (two wells/cAMP concentration).

Negative and positive control:

Each assay microtiter plate contains as negative controls 8 wells in column 23 with vehicle controls instead of compound (100 % CTL; 50-100 nM L-Glutamic acid + 0.03 µM forskolin + 0.5 mM IBMX + 1% DMSO); and 8 wells in column 23 as positive control (200% CTL; 100 µM L-Glutamic acid + 0.03 µM forskolin + 0.5 mM IBMX + 1% DMSO).

Calculation:

The analysis of the data is performed by the calculation of the ratio between the emission at 665 nm and the emission at 615 nm (Em665/Em615 ratio). Thereafter the signals of the compounds were normalized using the positive and negative controls by the following formula:

PoC = 100 + 100 x ((Negative Control - Signal Sample) / (Negative Control - Positive Control))

A potentiator of $mGlu_3$ receptor will give values between 100 %CTL (no activation) and 200%CTL (complete activation) after taking the variability of the assay into account. Values far outside this dynamic range of the assay are either due to compound/sample specific physicochemical properties (e.g. solubility, light absorbance, quenching, reactivity) which caninterfere with the assay technology or due to the fact that these compounds may act as a activators of the ion channel.

In vitro molecular pharmacology methods (GIRK)

$mGlu_3$ Thallium Flux Assay

Human embryonic kidney 293 (HEK293) cell lines stably co-expressing human or rat $mGlu_3$ and G protein-coupled inwardly rectifying potassium (GIRK) channels were maintained in growth medium containing DMEM/F12 containing 10% FBS, 20 mM HEPES, 1 mM sodium pyruvate, 2 mM L-glutamine, non-essential amino acids mixture, 100 units/ml penicillin/streptomycin, 700 µg/mL G418, and 0.6 µg/mL puromycin.

The day before the assay, $mGlu_3$ /GIRK cells were plated into 384 well, black-walled, clear-bottom poly-D-lysine coated plates at a density of 15,000 cells/20 µL/well in DMEM containing 10% dialyzed FBS, 20 mM HEPES, 1 mM sodium pyruvate, and 100 units/mL penicillin/streptomycin. The cells were incubated overnight at 37 °C in the presence of 5% CO_2.

The next day, GIRK assay buffer (Hank's balanced salt solution (HBSS), 20 mM HEPES, and 4.16 mM sodium bicarbonate, pH 7.4) was prepared and used to dilute compounds and Thallos-acetomethoxyester (Thallos-AM, Ion Biosciences), a fluorescent thallium indicator. Compounds were serially diluted 1:3 into 10-point concentration response curves in DMSO using a Bravo Liquid Handler (Agilent, Santa Clara, CA), transferred to a 384 well daughter plates using an Echo acoustic liquid handler (Beckman Coulter, Indianapolis, Indiana), and diluted in assay buffer to a 2X final concentration. A 2X dye solution (1.36 µM) was prepared by mixing a 2.97 mM Thallos-AM stock in DMSO with 10% (w/v) pluronic acid F-127 in a 1:1 ratio in assay buffer. The agonist plates were prepared using glutamate concentrations for the EC_{20} and EC_{Max} responses to a 5X final concentration in thallium stimulation buffer (125 mM $NaHCO_3$, 1.8 mM $CaSO_4$, 1 mM $MgSO_4$, 5 mM glucose, 12.5 mM Tl_2SO_4, 10 mM HEPES, pH 7.4). Using a microplate washer (BioTek, Winooski, VT), cells were washed with GIRK assay buffer 3 times to remove media. After the final wash, 20 µL of assay buffer remained in the cell plates. Immediately, 20 µL of the 2X dye solution (final 0.68 µM) was added to each well of the cell plate using a Multidrop Combi dispenser (Thermo Fisher, Waltham, MA). After cells were incubated with the dye solutions for 60 min at room temperature, the dye solutions were removed and replaced with GIRK assay buffer using a microplate washer, leaving 20 µL of assay buffer in the cell plate. The compound, agonist, and cell plates were placed inside the Functional Drug Screening System (FDSS 7000 or µCell kinetic imaging plate reader, Hamamatsu, Japan) to measure the thallium flux using a double add

protocol. After establishment of a fluorescence baseline (excitation, 480 nm; emission, 530 nm), 20 µL (2x) of test compound was added to the cells at 2 seconds and the response was measured. 140 seconds later, 10 µL (5x) of an EC_{20} concentration of glutamate or vehicle in thallium stimulus buffer was added to the cells, and the response of the cells was measured for an additional 158 seconds. Multiple reference wells containing no compound (DMSO vehicle) received either no glutamate (for a baseline reference), EC_{20} glutamate, or EC_{Max} glutamate (for normalization to maximum response). Fluorescence was measured throughout the experiment at a frequency of 1 measurement per 2 seconds before the glutamate/thallium addition and a frequency of 1 measurement per 1 second after the glutamate/thallium addition. Each compound concentration series occurred one time in each plate, and replicates from two or three plates were used in each experimental run. Thallium solutions and plastic solid waste were handled and disposed of according to guidelines from the Vanderbilt University Chemical Safety department. Data were normalized using a static ratio function (F/F_0) by dividing every fluorescent measurement by the initial fluorescent value for the corresponding well. The increase in signal resulting from the glutamate/thallium addition of the second add was measured by determining the slope from the time window of 145 seconds to 155 seconds. The average of all baseline slopes (no compound, no glutamate) was determined and this value was subtracted from all other slope values. The average of all EC_{Max} slopes was determined, and this value was used to normalize the baseline-corrected slopes to a percent max value ($\%E_{Max}$). Compound-evoked increases in thallium flux response in the presence of glutamate EC_{20} agonist were determined as potentiator activity, and potency (EC_{50}) and maximum responses (% Glu_{Max}) of compounds were determined using a four-parameter logistical equation using GraphPad Prism (La Jolla, CA) or the Dotmatics software platform (Woburn, MA):

$$y = bottom + \frac{top - bottom}{1 + 10^{(LogEC50 - A)Hillslope}}$$

where A is the molar concentration of the compound; bottom and top denote the lower and upper plateaus of the concentration-response curve; HillSlope is the Hill coefficient that describes the steepness of the curve; and EC_{50} is the molar concentration of compound required to generate a response halfway between the top and bottom.

In vitro and *in vivo* DMPK Methods

Plasma protein binding: The protein binding of each compound was determined in plasma via equilibrium dialysis employing RED Plates (ThermoFisher Scientific, Rochester, NY). Plasma was added to the 96 well plate containing test compound and mixed thoroughly for a final concentration of 5 μM. Subsequently, an aliquot of the plasma-compound mixture was transferred to the *cis* chamber (red) of the RED plate, with an phosphate buffer (25 mM, pH 7.4) in the *trans* chamber. The RED plate was sealed and incubated for 4 hours at 37°C with shaking. At completion, aliquots from each chamber were diluted 1:1 with either plasma (*cis*) or buffer (*trans*) and transferred to a new 96 well plate, at which time ice-cold acetonitrile containing internal standard (50 ng/mL carbamazepine) (2 volumes) was added to extract the matrices. The plate was centrifuged (3000 rcf, 10 min) and supernatants transferred and diluted 1:1 (supernatant: water) into a new 96 well plate, which was then sealed in preparation for LC/MS/MS analysis. Each compound was assayed in triplicate within the same 96-well plate. Fraction unbound was determined using the following equation

$$F_u = \frac{Conc_{buffer}}{Conc_{plasma}}$$

Intrinsic clearance: Human or rat hepatic microsomes (0.5 mg/mL) and 1 μM test compound were incubated in 100 mM potassium phosphate pH 7.4 buffer with 3 mM $MgCl_2$ at 37°C with constant shaking. After a 5 min preincubation, the reaction was initiated by addition of NADPH (1 mM). At selected time intervals (0, 3, 7, 15, 25, and 45 min), aliquots were taken and subsequently placed into a 96-well plate containing cold acetonitrile with internal standard (50 ng/mL carbamazepine). Plates were then centrifuged at 3000 rcf (4° C) for 10 min, and the supernatant was transferred to a separate 96-well plate and diluted 1:1 with water for LC/MS/MS analysis. The *in vitro* half-life ($T_{1/2}$, min, Eq. 1), intrinsic clearance (CL_{int}, mL/min/kg, Eq. 2) and subsequent predicted hepatic clearance (CL_{hep}, mL/min/kg, Eq. 3) was determined employing the following equations:

(1) $$T_{1/2} = \frac{Ln(2)}{k}$$

where k represents the slope from linear regression analysis of the natural log percent remaining

of test compound as a function of incubation time

(2) $$CL_{\text{int}} = \frac{0.693}{in\,vitro\,T_{1/2}}\, x\, \frac{mL\ incubation}{mg\ microsomes}\, x\, \frac{45\,mg\,microsomes}{gram\ liver}\, x\, \frac{20^{a}\ gram\ liver}{kg\ body\ wt}$$

[a]scale-up factors: of 20 (human) or 45 (rat)

(3) $$CL_{hep} = \frac{Q_h \cdot CL\,\text{int}}{Q_h + CL\,\text{int}}$$

where Q_h (hepatic blood flow, mL/min/kg) is 21 (human) or 70 (rat).

LC/MS/MS Bioanalysis of Samples from Plasma Protein Binding and Intrinsic Clearance Assays. Samples were analyzed on a Thermo Electron TSQ Quantum Ultra triple quad mass spectrometer (San Jose, CA) via electrospray ionization (ESI) with two Themo Electron Accella pumps (San Jose, CA), and a Leap Technologies CTC PAL autosampler (Carrboro, NC). Analytes were separated by gradient elution on a dual column system with two Thermo Hypersil Gold (2.1 x 30 mm, 1.9 µm) columns (San Jose, CA) thermostated at 40°C. HPLC mobile phase A was 0.1% formic acid in water and mobile phase B was 0.1% formic acid in acetonitrile. The gradient started at 10% B after a 0.2 min hold and was linearly increased to 95% B over 0.8 min; hold at 95% B for 0.2 min; returned to 10% B in 0.1 min. The total run time was 1.3 min and the HPLC flow rate was 0.8 mL/min. While pump 1 ran the gradient method, pump 2 equilibrated the alternate column isocratically at 10% B. Compound optimization, data collection and processing was performed using Thermo Electron's QuickQuan software (v2.3) and Xcalibur (v2.0.7 SP1).

Inhibition of Cytochrome P450 Enzymes: A cocktail of substrates for cytochrome P450 enzymes (1A2: Phenacetin, 10 µM; 2C9: Diclofenac, 5 µM; 2D6: Dextromethorphan, 5 µM; 3A4: Midazolam, 2 µM) were mixed for cocktail analysis. For P450 2C19, the substrate stock (Mephenytoin, 40 µM)and substrate mix were prepared separately for discrete analysis. The positive control for pan-P450 inhibition (miconazole) was included alongside each test compound in analysis.

A reaction mixture of 100 mM Kpi, pH 7.4, 0.1 mg/mL human liver microsomes (HLM) and Substrate Mix is prepared and aliquoted into a 96-deepwell block. Test compound and positive control (in duplicate) were then added such that the final concentration of test compound ranged

from 0.1 – 30 μM. The plate was vortexed briefly and then pre-incubated at 37°C while shaking for 15 minutes. The reaction was initiated with the addition of NADPH (1 mM final concentration). The incubation continued for 8 min and the reaction quenched by 2x volume of cold acetonitrile containing internal standard (50 nM carbamazepine). The plate was centrifuged for 10 minutes (4000 rcf, 4°C) and the resulting supernatant diluted 1:1 with water for LC/MS/MS analysis. A 12 point standard curve of substrate metabolites over the range of 0.98 nM to 2000 nM.

Samples were analyzed via electrospray ionization (ESI) on an AB Sciex API-4000 (Foster City, CA) triple-quadrupole instrument that was coupled with Shimadzu LC-10AD pumps (Columbia, MD) and a Leap Technologies CTC PAL auto-sampler (Carrboro, NC). Analytes were separated by gradient elution using a Fortis C18 3.0 x 50 mm, 3 μm column (Fortis Technologies Ltd, Cheshire, UK) thermostated at 40°C. HPLC mobile phase A was 0.1% formic acid in water (pH unadjusted), mobile phase B was 0.1% formic acid in acetonitrile (pH unadjusted). The gradient started at 10% B after a 0.2 min hold and was linearly increased to 90% B over 1.2 min; held at 90% B for 0.1 min and returned to 10% B in 0.1 min followed by a re-equilibration (0.9 min). The total run time was 2.5 min and the HPLC flow rate was 0.5 mL/min. The source temperature was set at 500°C and mass spectral analyses were performed using multiple reaction monitoring (MRM), with transitions specific for each compound utilizing a Turbo-Ionspray® source in positive ionization mode (5.0 kV spray voltage).

The IC_{50} values for each compound were obtained for the individual CYP enzymes by quantitating the inhibition of metabolite formation for each probe substrate. A 0 μM compound condition (or control) was set to 100% enzymatic activity and the effect of increasing test compound concentrations on enzymatic activity could then be calculated from the % of control activity. Curves were fitted using XLfit 5.2.2 (four-parameter logistic model, equation 201) to determine the concentration that produces half-maximal inhibition (IC_{50}).

In vivo DMPK experimental

Compounds were formulated as 10% tween 80 micro suspensions in sterile water at the concentration of 1 mg/ml and administered intraperitoneally to male Sprague- Dawley rats weighing 225 to 250 g (Harlan, Inc., Indianapolis, IN) at the dose of 10 mg/kg. The rat blood and brain were collected at 0.25 hr. Animals were euthanized and decapitated, and the brains were

removed, thoroughly washed in cold phosphate buffered saline and immediately frozen on dry ice. Trunk blood was collected in EDTA Vacutainer tubes, and plasma was separated by centrifugation and stored at -80°C until analysis. Plasma was separated by centrifugation (4000 rcf, 4°C) and stored at 80°C until analysis. On the day of analysis, frozen whole-rat brains were weighed and diluted with 1:3 (w/w) parts of 70:30 isopropanol:water. The mixture was then subjected to mechanical homogenation employing a Mini-Beadbeater™ and 1.0 mm Zirconia/Silica Beads (BioSpec Products) followed by centrifugation. The sample extraction of plasma (20 µL) or brain homogenate (20 µL) was performed by a method based on protein precipitation using three volumes of ice-cold acetonitrile containing an internal standard (50 ng/mL carbamazepine). The samples were centrifuged (3000 rcf, 5 min) and supernatants transferred and diluted 1:1 (supernatant: water) into a new 96 well plate, which was then sealed in preparation for LC/MS/MS analysis.

In vivo samples were analyzed via electrospray ionization (ESI) on an AB Sciex API-5500 QTrap (Foster City, CA) instrument that was coupled with Shimadzu LC-20AD pumps (Columbia, MD) and a Leap Technologies CTC PAL auto-sampler (Carrboro, NC). Analytes were separated by gradient elution using a Fortis C18 3.0 x 50 mm, 3 µm column (Fortis Technologies Ltd, Cheshire, UK) thermostated at 40°C. HPLC mobile phase A was 0.1% formic acid in water (pH unadjusted), mobile phase B was 0.1% formic acid in acetonitrile (pH unadjusted). The gradient started at 30% B after a 0.2 min hold and was linearly increased to 90% B over 0.8 min; held at 90% B for 0.5 min and returned to 30% B in 0.1 min followed by a re-equilibration (0.9 min). The total run time was 2.5 min and the HPLC flow rate was 0.5 mL/min. The source temperature was set at 500°C and mass spectral analyses were performed using multiple reaction monitoring (MRM), with transitions specific for each compound utilizing a Turbo-Ionspray® source in positive ionization mode (5.0 kV spray voltage). The calibration curves were constructed in blank plasma. All data were analyzed using AB Sciex Analyst software v1.5.1.

Behavioral Pharmacology Methods

Animals: Adult male Sprague-Dawley rats (Envigo, Inc., Indianapolis, IN) were used. They were housed in the animal care facility certified by the American Association for the Accreditation of Laboratory Animal Care (AALAC) under a 12-hour light/dark cycle (lights on: 7 a.m.; lights off: 7 p.m.) and had free access to food and water. The experimental protocols performed during the light cycle were approved by the Institutional Animals Care and Use Committee of Vanderbilt University and conformed to the guidelines established by the National Research Council Guide for the Care and Use of Laboratory Animals.

Novel Object Recognition: The rats were habituated for 10 minutes to the novel object recognition (NOR) arenas for two consecutive days prior to testing. The NOR arena consisted of a dark-colored plexiglass box (40 X 64 X 33 cm) and was cleaned with 70% ethanol between animals. In one set of experiments, on day 3 the rats were habituated to the dosing room for 2 hours and then administered vehicle (0.5% natrasol/0.015% Tween 80) or VU6052959 (3-30 mg/kg p.o., 10mL/kg, n=19-20) and were placed back into their homecage for 60 min. In a second set of experiments, on day 3 the rats were dosed with vehicle or VU6052959 (30 mg/kg p.o. 10mL/kg, n=14-15) either and placed back in their homecage for 14 hrs or 60 min. In a third set of experiments, the rats were either subchronically or acutely administered vehicle or VU6052959 (30 mg/kg p.o. 10 mL/kg n=12-14) as shown in **Figure 7** with the rats returning to their homecage for 24 hrs or 60 min after the final dosing. The rats were then placed into the NOR arena with 2 identical objects for ten minutes and then placed back into the home cage. The rats were then placed back into the NOR arena with one of the familiar objects replaced with a novel object at 24 hrs after the familiar object exposure. The activity of the animals was recorded for 5 min and the time spent exploring each of the objects is scored by a blinded observer. Recognition index was calculated as [(time spent exploring novel object) – (time spent exploring familiar object)]/total time exploring objects.

Reversal of Scopolamine or MK-801 induced deficits in NOR: The rats were habituated for 10 minutes to the novel object recognition (NOR) arenas for one day prior to testing. The NOR arena consisted of a dark-colored plexiglass box (40 X 64 X 33 cm) and was cleaned with 70% ethanol between animals. On day 2 the rats were habituated to the dosing room for 2 hours and then

administered vehicle (0.5% natrasol/0.015% Tween 80) or VU6052959 (3-30 mg/kg p.o., 10mL/kg, n=7-12) and were placed back into their homecage for 60 min. The rats were then administered vehicle, scopolamine (1 mg/kg i.p. 1 ml/kg; 10% Tween 80) or MK-801 (0.075 mg/kg s.c. 1 ml/kg; saline) and placed back in their homecage for 30 min. The rats were then placed into the NOR arena with 2 identical objects for ten minutes and then placed back into the home cage. The rats were then placed back into the NOR arena with one of the familiar objects replaced with a novel object at 2 hrs after the familiar object exposure. The activity of the animals was recorded for 5 min and the time spent exploring each of the objects is scored by a blinded observer. Recognition index was calculated as [(time spent exploring novel object) – (time spent exploring familiar object)]/total time exploring objects.
